# Combined transcriptomic and metabolomic analysis of the mechanism by which *Bacillus velezensis* induces resistance to anthracnose in walnut

**DOI:** 10.3389/fmicb.2024.1420922

**Published:** 2024-10-09

**Authors:** Linmin Wang, Tianhui Zhu

**Affiliations:** ^1^College of Forestry, Sichuan Agricultural University, Chengdu, Sichuan, China; ^2^School of Agronomy and Horticulture, Chengdu Agricultural College, Chengdu, Sichuan, China; ^3^National Forestry and Grassland Administration Key Laboratory of Forest Resources Conservation and Ecological Safety on the Upper Reaches of the Yangtze River, Chengdu, China

**Keywords:** *Bacillus velezensis*, *Colletotrichum gloeosporioides*, metabolome, transcriptome, induced resistance

## Abstract

Walnut (*Juglans* spp.), a significant deciduous tree of economic and ecological importance, faces substantial threats from walnut anthracnose, primarily caused by *Colletotrichum gloeosporioides*. *Bacillus velezensis* has shown promise in mitigating this fungal pathogen. To delve deeper into the induction mechanism of *B. velezensis* on walnut plant resistance, we conducted a metabolomic analysis on walnut leaves from six different treatment groups. Specifically, the groups were defined as follows: Group B.v. was inoculated with *B. velezensis* alone, Group CK served as the blank control, and Group C.g. was inoculated solely with *C. gloeosporioides*. Group B.v.−C.g. received *B. velezensis* followed by *C. gloeosporioides* inoculation. Group B.v.+C.g. underwent simultaneous inoculation with both *B. velezensis* and *C. gloeosporioides*, while Group C.g.−B.v. was treated first with *C. gloeosporioides* then *B. velezensis*. A total of 1,503 metabolites were detected, mainly including flavonoids, terpenoids, and steroids. The results revealed that *B. velezensis* spraying not only enhanced the inherent resistance of walnut plants but also significantly regulated walnut plants already infected with *C. gloeosporioides*. This was mainly achieved by inducing walnut plants to adjust their metabolic pathways such as salicylic acid, jasmonic acid, and abscisic acid, thereby strengthening their stress response. Transcriptomic and metabolomic correlation analyses showed that in the comparisons of B.v. vs. CK, C.g. vs. CK, and C.g.−B.v. vs. C.g., 59, 244, and 122 differential abundance metabolites were detected, along with 7860, 3677, and 5587 differential genes, respectively. Amino acid synthesis, starch and sucrose metabolism, photosynthesis, phenylpropane metabolism, purine metabolism, and glutathione metabolism played crucial roles in walnut’s disease resistance mechanism. Further analysis revealed that *B. velezensis* induced walnut plants to regulate multiple genes, such as *LOC109005403*, *LOC108985444* and *LOC118344177*, resulting in the production of defensive metabolites such as palmitic acid, coumarin and ferulic acid, thereby enhancing their resistance to *C. gloeosporioides*. In summary, *B. velezensis* induces systemic resistance in walnut plants by modulating the metabolic pathways of salicylic acid, jasmonic acid, and abscisic acid. It enhances this resistance by strengthening cell walls, synthesizing defensive secondary metabolites, and regulating energy metabolism and stress responses. These findings provide a solid theoretical foundation for the future field application of *B. velezensis* in controlling walnut anthracnose.

## 1 Introduction

In the face of microbial and abiotic threats, plants have evolved a variety of defense mechanisms, with Induced Systemic Resistance (ISR) playing a pivotal role (Pieterse et al., 1998; Doornbos et al., 2011; Rania et al., 2017; Sahebani and Omranzade, 2020; Saxena et al., 2020). ISR is not an immediate reaction but a sensitization process that prepares plants for rapid and robust responses to potential threats. Key players in ISR include various *Bacillus* species, which as plant endophytes can trigger ISR when they reach a certain population density within the host (Beneduzi et al., 2012; Pieterse et al., 2014; Wu et al., 2018). The effectiveness of ISR induction by *Bacillus* strains, however, varies, depending on the application method and treatment intervals (Zhang et al., 2002). *Bacillus* spp. and their metabolites enhance plant defenses by stimulating pathogenesis-related (PR) protein genes and defense-related enzymes (Ali et al., 2022; Chouhan et al., 2023). For instance, *Bacillus amyloliquefaciens* Ba13 increased resistance to tomato yellow leaf curl virus by altering the microbial structure of the rhizosphere (Guo et al., 2019). Similarly, a study by Fu et al. (2020) revealed that foliar application of an endophytic *Bacillus* strain controlled tomato bacterial wilt by upregulating *PR-1a* and *LoxD* genes. Additionally, Rania et al. (2017) found that *Bacillus* spp. from wild *Solanaceous* species upregulate PR and lipoxygenase genes in tomatoes, enhancing resistance to *Fusarium oxysporum*. *B. velezensis* S3-1 boosts wilt resistance in pepper plants through the upregulation of defense genes *CaPR1* and *CaPIN II* (Fan et al., 2023b). The molecular basis of ISR involves specific signal transduction pathways, predominantly those governed by plant hormones such as jasmonic acid (JA) and ethylene (ET) (Pieterse et al., 1998; Hossain et al., 2017; Nie et al., 2017; Romera et al., 2019). A prime example is the *Bacillus licheniformis* strain CH102, which boosts the host plant’s JA pathway, thereby enhancing resistance to fungal pathogens (Sukkasem et al., 2018). *B. amyloliquefaciens* YN201732 induces systemic resistance in tobacco against powdery mildew, evidenced by activation of the JA/ET signaling pathway (Jiao et al., 2020). Similarly, brief exposure to volatile compounds from *B. subtilis* GB03 and *B. amyloliquefaciens* IN937a has been shown to activate ISR in *Arabidopsis thaliana*, primarily via the ET pathway and independent of the salicylic (SA) and JA pathways (Ryu et al., 2004). Further, *Bacillus* species are known to modulate host plant enzyme activities and related pathways to fortify plant defenses. For instance, *B. licheniformis* W10 has been found to regulate ROS levels and activate defense-related enzymes, thereby bolstering resistance against brown rot (Ji et al., 2020). *Bacillus cereus*-Amazcala (B.c-A) is another example, known for stimulating CAT and PAL activities in pepper plants, leading to enhanced immune responses (Ferrusquía-Jiménez et al., 2022). Crucially, Samaniego-Gámez et al. (2021) have shown that inoculation with specific *Bacillus* spp. can boost photosynthetic efficiency and ISR in *Capsicum chinense* plants, offering protection against pepper golden mosaic virus and improving plant health and fruit yield. Similarly, Chen et al. (2009) discovered that *B. amyloliquefaciens*-derived elicitors can induce resistance to various pathogens in soybean and tomato plants through multiple defense signaling pathways, with surfactin and Fengycin playing significant roles as inducers. In conclusion, *Bacillus*, as an endophytic bacterium, plays a vital role in stimulating plant innate immunity and reinforcing defense mechanisms against pathogens.

Walnut was a valuable agricultural commodity, but its production was threatened by walnut anthracnose, primarily caused by *C. gloeosporioides* (Wang et al., 2020; Fang et al., 2021; Feng et al., 2021). This disease disrupted the growth and reduced the economic value of walnut trees. Biological management offered a sustainable solution that protected ecosystems and human health while effectively controlling this pathogen (He et al., 2021). *Bacillus* spp., notably *B. velezensis*, serve as key agents in biocontrol, effectively mitigating numerous fungal infections (Wang et al., 2023, 2024; Wei et al., 2023; Armenova et al., 2024; Fessia et al., 2024; Russi et al., 2024). Esteemed for its biocontrol proficiency, *B. velezensis* has become a focal point of research due to its capacity to combat diverse diseases including tomato early blight, maize leaf blight, and pepper anthracnose (da Silva Junior et al., 2023; Rahman et al., 2023; Zhou et al., 2023). Additionally, it plays a crucial role in fostering the growth of crops such as cotton, rice, and tomatoes (Balderas-Ruíz et al., 2021; Do et al., 2023; Tang et al., 2023). In our previous research, we discovered that *B. velezensis* exhibits a significant antagonistic effect against *C. gloeosporioides*. This interaction was demonstrated by a notable reduction in the area of lesions caused by *C. gloeosporioides* on detached walnut leaves (Wang and Zhu, 2023). In this research, pot experiments were conducted to evaluate the effects of inoculating *B. velezensis* at various time points on the control of walnut anthracnose. To elucidate the mechanisms of ISR in walnut plants mediated by *B. velezensis*, transcriptomic and metabolomic analyses of walnut leaves were performed. These analyses detailed how *B. velezensis* regulates gene expression and influences metabolic pathways and the secretion of resistance compounds, providing a theoretical basis for its future production and development.

## 2 Materials and methods

### 2.1 Setup of walnut potted plant experiment

Walnut plants used in this study were 5-year-old trees of the “Xiangling” variety, grown at the Chengdu academy of agriculture and forestry sciences in Chengdu, Sichuan province, China, located at coordinates 104.07275 longitude, 30.57899 latitude, and an elevation of 534 meters. The experimental design consisted of six distinct groups to assess the interactions and effects of *B. velezensis* and *C. gloeosporioides* on walnut plants. Group B.v. received inoculation with *B. velezensis* alone, while Group CK served as the blank control. Group C.g. was inoculated solely with *C. gloeosporioides*, while Group B.v.−C.g. received *B. velezensis* followed by *C. gloeosporioides*. Group B.v.+C.g. was subjected to simultaneous inoculation with *B. velezensis* and *C. gloeosporioides*, while Group C.g.−B.v. was inoculated with *C. gloeosporioides* followed by *B. velezensis*. Each group comprised 15 walnut trees, totaling 90 trees. These trees had an average height of 1.1 meters, with no significant difference in trunk diameter. The selected walnut plants were uniform in size and closely matched in growth height to ensure uniform physiological states and growth stages across all groups at the start of the experiment. Transparent plastic film was used to isolate each group of walnut trees, thereby preventing cross-contamination and ensuring the accuracy and reliability of the experiment. However, it should be noted that this experimental setup did not employ a randomized block design.

To prepare the spore suspension of *C. gloeosporioides* (preserved at the laboratory of forest protection, college of forestry, Sichuan agricultural university), *C. gloeosporioides* cakes were inoculated onto PDA solid medium and then incubated at a constant temperature of 28°C for 10 days. The surface of the culture medium was repeatedly washed with sterile water to evenly disperse the conidia in the water. The resulting conidial suspension was filtered through double-layer gauze and counted under an optical microscope using a hemocytometer to adjust the concentration to 1 × 10^6^ spores/mL. Following the protocol outlined by Wang and Zhu (2023), a 100-fold concentrated fermentation liquid of *B. velezensis* (preserved at the Laboratory of Forest Protection, College of Forestry, Sichuan Agricultural University) was prepared and administered to the designated groups of walnut plants every 3 days via spraying and root irrigation. Each tree received 500 mL of the solution at the root. The spray was applied uniformly on the leaves and stems of the walnut plants with consistent spray pressure and droplet size, ensuring the liquid covered the plant surfaces without dripping. Detailed treatment methods and scheduling are provided in Supplementary Table 1.

Following the inoculation with *C. gloeosporioides*, regular observations were conducted every 15 days, starting from day 15, for a total of four observations. Based on the observation results, disease incidence was recorded, and the disease incidence rate as well as the relative control efficacy of *B. velezensis* against *C. gloeosporioides* were calculated. To ensure data reliability, each group of walnut plants was randomly numbered, with five walnut plants forming one replicate, and three replicates in total.

Formula (1): Disease incidence rate


(1) $$\mathrm{Disease}\mathrm{incidence}\mathrm{rate}\left(\%\right)=\left(\frac{N_{d}}{N_{t}}\right)\times 100$$

*N*_*d*_: Number of diseased plants; *Nt*: Total number of inoculated plants.

Formula (2): Relative control effect


(2) $$\mathrm{Relative}\mathrm{control}\mathrm{effect}\left(\%\right)=\left(\frac{I_{c}-I_{t}}{I_{c}}\right)\times 100$$

*Ic*: Disease index of control; *It*: Disease index of treatment.

On the 40th day of the pot experiment, six walnut trees were randomly selected from each treatment group to collect uniform-sized leaves from the same positions for metabolomic analysis, with six replicates per treatment group. The leaves were immediately labeled and flash-frozen in liquid nitrogen for storage. Non-targeted metabolomics techniques were utilized for the metabolomic analysis.

### 2.2 Untargeted metabolomics

#### 2.2.1 Sample preparation for UHPLC-MS/MS

Tissues (100 mg) were individually grounded with liquid nitrogen and the homogenate was resuspended with prechilled 80% methanol by well vortex. The samples were incubated on ice for 5 min and then were centrifuged at 15,000 *g*, 4°C for 20 min. Some of supernatant was diluted to final concentration containing 53% methanol by LC-MS grade water. The samples were subsequently transferred to a fresh Eppendorf tube and then were centrifuged at 15,000 *g*, 4°C for 20 min. Finally, the supernatant was injected into the LC-MS/MS system analysis (Want et al., 2013).

#### 2.2.2 UHPLC-MS/MS analysis

UHPLC-MS/MS analyses were performed using a Vanquish UHPLC system (Thermo Fisher, Germany) coupled with an Orbitrap Q Exactive™ HF mass spectrometer (Thermo Fisher, Germany) in Novogene Co., Ltd (Beijing, China). Samples were injected onto a Hypesil Gold column (100 × 2.1 mm, 1.9 μm) using a 12-min linear gradient at a flow rate of 0.2 mL/min. The eluents for the positive polarity mode were eluent A (0.1% FA in Water) and eluent B (Methanol). The eluents for the negative polarity mode were eluent A (5 mM ammonium acetate, pH 9.0) and eluent B(Methanol). The solvent gradient was set as follows: 2% B, 1.5 min; 2–85% B, 3 min; 85–100% B, 10 min;100–2% B, 10.1 min;2% B, 12 min. Q Exactive™ HF mass spectrometer was operated in positive/negative polarity mode with spray voltage of 3.5 kV, capillary temperature of 320°C, sheath gas flow rate of 35 psi and aux gas flow rate of 10 L/min, S-lens RF level of 60, Aux gas heater temperature of 350°C.

#### 2.2.3 Data processing and metabolite identification

The raw data files generated by UHPLC-MS/MS were processed using the Compound Discoverer 3.1 (CD3.1, Thermo Fisher) to perform peak alignment, peak picking, and quantitation for each metabolite. Subsequently, peak alignment across different samples was performed using a retention time deviation of 0.2 min and a mass deviation of 5 ppm to enhance identification accuracy. Following this, peak extraction was conducted with criteria including a mass deviation of 5 ppm, signal intensity deviation of 30%, signal-to-noise ratio of 3, minimum signal intensity, and summation of ion intensities. Quantification was carried out based on peak areas, integrating target ions. Molecular formula prediction utilized molecular and fragment ions, comparing results against the mzCloud,^1^ mzVault, and Masslist databases. Background ions were removed using blank samples, and raw quantitative results were subsequently standardized. Finally, metabolite identification and relative quantification results were obtained. Data processing was conducted using the Linux operating system (CentOS version 6.6) and software such as R(R version R-3.4.3) and Python (Python 2.7.6 version). When data were not normally distributed, normal transformations were attempted using of area normalization method.

#### 2.2.4 Data analysis

These metabolites were annotated using the KEGG database,^2^ HMDB database^3^ and LIPIDMaps database^4^. Principal components analysis (PCA) and Partial least squares discriminant analysis (PLS-DA) were performed at metaX (Wen et al., 2017) (a flexible and comprehensive software for processing metabolomics data). We applied univariate analysis (*t*-test) to calculate the statistical significance (*P*-value). The metabolites with VIP > 1 and *P*-value < 0.05 and fold change ≥ 2 or FC ≤ 0.5 were considered to be differential abundance metabolites. Volcano plots were used to filter metabolites of interest which based on log_2_(FoldChange) and -log_10_(*p*-value) of metabolites by ggplot2 in R language. For clustering heat maps, the data were normalized using z-scores of the intensity areas of differential abundance metabolites and were ploted by Pheatmap package in R language. The correlation between differential abundance metabolites were analyzed by cor () in R language (method = pearson). Statistically significant of correlation between differential abundance metabolites were calculated by cor.mtest() in R language. *P*-value < 0.05 was considered as statistically significant and correlation plots were ploted by corrplot package in R language. The functions of these metabolites and metabolic pathways were studied using the KEGG database. The metabolic pathways enrichment of differential abundance metabolites was performed, when ratio was satisfied by x/n > y/N, metabolic pathway was considered as enrichment, when *P*-value of metabolic pathway < 0.05, metabolic pathway was considered as statistically significant enrichment.

### 2.3 Transcriptome sequencing methods

#### 2.3.1 Procedures for RNA extraction, sequencing library construction, and sequencing

Total RNA was extracted from walnut leaves using TRIzol Reagent (Invitrogen, cat. no. 15596026) as per the protocol described by Chomczynski and Sacchi (1987). Post-extraction, the RNA samples were treated with DNase I to remove any contaminating DNA. The purity and quality of the extracted RNA were assessed using the Nanodrop™ OneC spectrophotometer (Thermo Fisher Scientific Inc.), focusing on the A260/A280 absorbance ratio. Additionally, RNA integrity was verified through 1.5% agarose gel electrophoresis. Quantification of the RNA was performed using the Qubit™ 3.0 Fluorometer with the RNA Broad Range Assay kit (Life Technologies, cat. no. Q10210). For the library construction, 2 μg of total RNA was used to prepare stranded RNA sequencing libraries using the KC-Digital™ Stranded mRNA Library Prep Kit for Illumina^®^ (Catalog NO. DR08502, Wuhan Seqhealth Co., Ltd., China). This kit incorporates a unique molecular identifier (UMI) of 8 random bases to minimize duplication bias during PCR amplification and sequencing. Following the manufacturer’s instructions, the library fragments ranging from 200 to 500 bps were enriched and quantified. The final library was sequenced on an Illumina NovaSeq 6000 system using the PE150 configuration.

#### 2.3.2 Quality control and data analysis

The raw sequencing data was initially processed using Trimmomatic (version 0.36) to filter out low-quality reads and trim adaptor-contaminated sequences. Subsequently, an in-house script was employed to address duplication bias inherent in the library preparation and sequencing steps. Specifically, clean reads were clustered based on their unique molecular identifier (UMI) sequences. Within each cluster, reads sharing the same UMI sequence were aligned pairwise, and those with sequence identity exceeding 95% were grouped into a new sub-cluster. Multiple sequence alignments within these sub-clusters facilitated the generation of a single consensus sequence per sub-cluster, effectively mitigating errors and biases introduced during PCR amplification and sequencing. These de-duplicated consensus sequences were then utilized for standard RNA-seq analysis. Mapping to the reference genome was performed using the STAR software (version 2.5.3a) with default settings. The reference genome for *Juglans regia* was sourced from the National Center for Biotechnology Information (NCBI) under BioProject ID PRJNA291087. Read counts corresponding to the exon regions of each gene were obtained using featureCounts (Subread-1.5.1; Bioconductor), and the relative expression levels were quantified as Reads Per Kilobase of transcript, per Million mapped reads (RPKM). Differential gene expression analysis between the groups was conducted using the edgeR package (version 3.12.1). Statistical significance was determined using a *p*-value cutoff of 0.05 and a fold-change threshold of 2, enabling the identification of genes with significant expression differences.

### 2.4 Metabolomic and transcriptomic association analysis

The transcriptomic and metabolomic analyses included four groups: Group B.v., inoculated solely with *B. velezensis*; Group CK, serving as the blank control; Group C.g., inoculated solely with *C. gloeosporioides*; and Group C.g.−B.v., where inoculation with *C. gloeosporioides* was followed by *B. velezensis*. The transcriptomic data has been uploaded to the NCBI database under accession number PRJNA1030780. To ensure data comparability and accuracy, a standardized sample collection method was employed, with specific timing and methods outlined in Section “2.1 Setup of walnut potted plant experiment.” Samples for both transcriptomic and metabolomic analyses were obtained from the same batch of walnut plants’ corresponding leaves, establishing a one-to-one correspondence. This approach aimed to minimize the impact of plant variations and environmental factors on the experimental results, thereby ensuring that the analysis accurately reflects the true relationship between gene expression and metabolite production. Metabolomic and transcriptomic KEGG enrichment analyses were conducted based on the same comparisons. By aligning the results, KEGG pathways enriched in both metabolomic and transcriptomic analyses were identified. Subsequently, a bubble plot illustrating the co-enriched pathways in metabolism and transcription was generated using the ggplot2 package in R language. This visualization method aids in comprehensively presenting the shared enriched pathways between metabolism and transcription.

## 3 Results

### 3.1 The Effect of *B. velezensis* application at different stages on anthracnose disease in potted walnut plants

**FIGURE 1:**
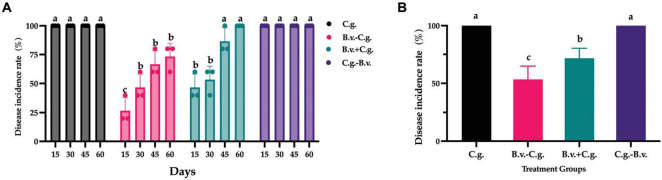
Statistical analysis results of disease incidence rates in different treatment groups. **(A)** Incidence rate statistical analysis results for three different groups across four distinct time points. Data were collected from 4 time points and 4 treatment groups. Analysis was conducted using one-way ANOVA and Duncan’s multiple range test in SPSS 16.0. The values depicted in the figure represent the average from three replicate experiments. Different lowercase letters indicate significant differences in incidence rates between treatments at the same time point (*P* < 0.05). **(B)** Estimated marginal mean values of incidence rates for different groups at various time points. Different letters signify significant differences between treatments based on repeated measures ANOVA (*P* < 0.05). Group C.g.: solely inoculated with *C. gloeosporioides*; Group B.v.–C.g.: *B. velezensis* first, then *C. gloeosporioides*; Group B.v.+C.g.: simultaneous inoculation; Group C.g.–B.v.: *C. gloeosporioides* first, then *B. velezensis*.

Based on observations and records at four time points, a statistical analysis of the incidence rate was conducted. The analysis results (Figure 1A) showed that by day 15, all walnut plants in groups C.g. and C.g.−B.v. exhibited disease symptoms, reaching an incidence rate of 100%. The incidence rates for treatment groups B.v.−C.g. and B.v.+C.g. gradually increased over time. By day 60, all walnut plants in group B.v.+C.g. showed disease symptoms, with an incidence rate of 100%, while the incidence rate for group B.v.−C.g. was 73.333%. The results preliminarily suggested an upward trend in the incidence rates of walnut plants following different treatments. However, to determine the statistical significance of this trend, a repeated measures analysis of variance (ANOVA) was required. The data passed the Levene’s test for homogeneity of variances, meeting the prerequisites for variance homogeneity, allowing for the selection of a repeated measures ANOVA to study the significance of changes in incidence rates. However, the data failed the sphericity test (*P* = 0.02 < 0.05), indicating asymmetry, thus the results of the multivariate tests were considered. According to the multivariate results, the time effect of Roy’s largest root was significant (*F*_(4,17)_ = 765.722, *P* < 0.05), indicating significant changes in the incidence rates of walnut plants over time. The time*treatment interaction effect of Roy’s largest root was also significant [*F*_(4,19)_ = 113.605, *P* < 0.05], suggesting an interaction effect between time points and treatment methods. Further repeated measures statistical analysis (Figure 1B) revealed that groups C.g. and C.g.−B.v. had the highest incidence rates, showing significant differences from the other two treatment groups. Group B.v.−C.g. had the lowest incidence rate, also significantly differing from the other three groups. For the three groups treated with *B. velezensis*, the incidence rates progressively increased from group B.v.−C.g. to group C.g.−B.v. The experimental results indicated that treating diseased walnut plants with *B. velezensis* at different stages significantly affected incidence rates of walnut plants. Notably, groups B.v.−C.g. and B.v.+C.g. successfully reduced the incidence rates of walnut plants to varying degrees.

**FIGURE 2:**
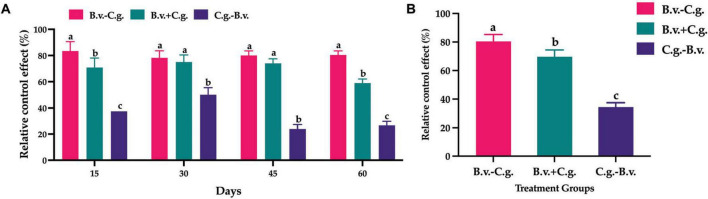
Statistical analysis of relative control effect among different treatment groups. **(A)** Relative control effect at four time points across three treatments, analyzed using one-way ANOVA and Duncan’s multiple range test (SPSS 16.0). The values in the figure represent the means of three repeated experiments, with different lowercase letters indicating significant differences in relative control effect among different treatment groups at the same time point (*P* < 0.05). Group B.v.–C.g.: *B. velezensis* first, then *C. gloeosporioides*; Group B.v.+C.g.: simultaneous inoculation; Group C.g.–B.v.: *C. gloeosporioides* first, then *B. velezensis*. **(B)** Estimated marginal means of relative control effect at different time points among different treatment groups. Different letters indicate significant differences (*P* < 0.05) based on repeated measures ANOVA.

The analysis of relative control effect (Figure 2A) revealed that groups B.v.−C.g., B.v.+C.g., and C.g.−B.v. demonstrated varying levels of disease control across the four observed time points, ranging from 22.00 to 87.50%. Among them, group B.v.−C.g. exhibited the best relative control effect, followed by group B.v.+C.g. Further statistical analysis on the population quantity of *B. velezensis* was conducted using the repeated measures analysis of variance (ANOVA) method. The data passed Levene’s test for homogeneity of variances, indicating that the study’s data met the prerequisites for homogeneity of variance, thus allowing the selection of a repeated measures ANOVA to study the significance of the trends in relative control effect. The data also passed the sphericity test (*P* = 0.425 > 0.05), indicating symmetry, hence the results were based on within-subject effects. According to the within-subject effects test results, the time effect was significant [*F*_(1,3)_ = 10.857, *P* < 0.05], meaning there was a significant change in control effectiveness over time. The interaction effect between time points and treatment methods was also significant [*F*_(3,9)_ = 29.828, *P* < 0.05], suggesting an interaction between time points and treatment methods. Further analysis of the repeated measures statistics (Figure 2B) showed that the three treatment groups exhibited different levels of relative control effect. Group C.g. achieved the best relative control effect, reaching 80.454%, which was more than twice that of group C.g.−B.v. The relative control effect of group C.g.−B.v. was lower, but its mean value also reached 34.512%. The comprehensive results indicated that treating walnut plants with *B. velezensis* at different time points improved relative control effect, particularly notable in groups B.v.−C.g. and B.v.+C.g., which showed commendable outcomes.

### 3.2 Metabolome data analysis

#### 3.2.1 Data quality control

The metabolomics data have been uploaded to the MetaboLights platform with the accession number MTBLS10608. The Pearson correlation coefficient between QC samples was calculated based on the relative quantitative values of metabolites (Rao et al., 2016). It can be seen from the results (Supplementary Figures 1 A, B) that the correlation of QC samples is high (close to 1), indicating that the whole detection process has good stability and high data quality. The PCA analysis of walnut leaf metabolites (Supplementary Figures 1 C, D) showed clear separation among the treatment groups. In the negative ion mode, PC1 and PC2 accounted for 13.08 and 11.54% of the variance, respectively. In the positive ion mode, PC1 and PC2 explained 14.45 and 9.68% of the variance. The tight clustering of QC samples indicated high data reliability.

#### 3.2.2 Metabolite pathway and classification annotation

**FIGURE 3:**
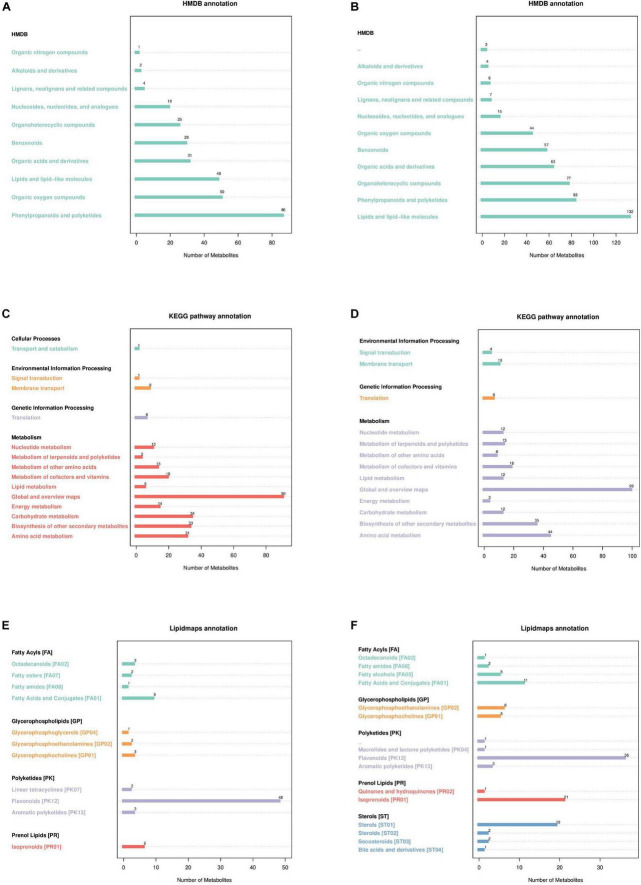
Annotated diagram of metabolite pathway and classification. **(A,B)** Annotated HMDB taxonomic map of metabolites. **(C,D)** Annotated map of the KEGG pathway of metabolites. **(E,F)** Annotated map of LIPID MAPS classification of metabolites.

According to the Human Metabolome Database (HMDB) in the neg and pos modes (Figures 3A, B), 86 and 83 metabolites correspond to phenylpropanoids and polyketides, 48 and 132 metabolites correspond to lipids and lipid-like molecules, 50 and 44 metabolites correspond to organic oxygen compounds, 31 and 63 metabolites correspond to organic acids and derivatives, and 25 and 77 metabolites correspond to organoheterocyclic compounds. The metabolites identified by the secondary profiles were subjected to the KEGG website for metabolic pathway analysis. In the neg and pos modes (Figures 3C, D), most metabolites were mainly annotated to the global and overview maps, carbohydrate metabolism, biosynthesis of other secondary metabolites and amino acid metabolism. LIPID MAPS annotation was performed on the identified metabolites, and the results showed that in the neg and pos modes (Figures 3E, F), the metabolites were mainly enriched in flavonoids, isoprenoids and steroids. Of these, 48 and 36 metabolites correspond to flavonoids, 6 and 21 metabolites correspond to isoprenoids.

#### 3.2.3 Analysis of differential metabolite expression

**FIGURE 4:**
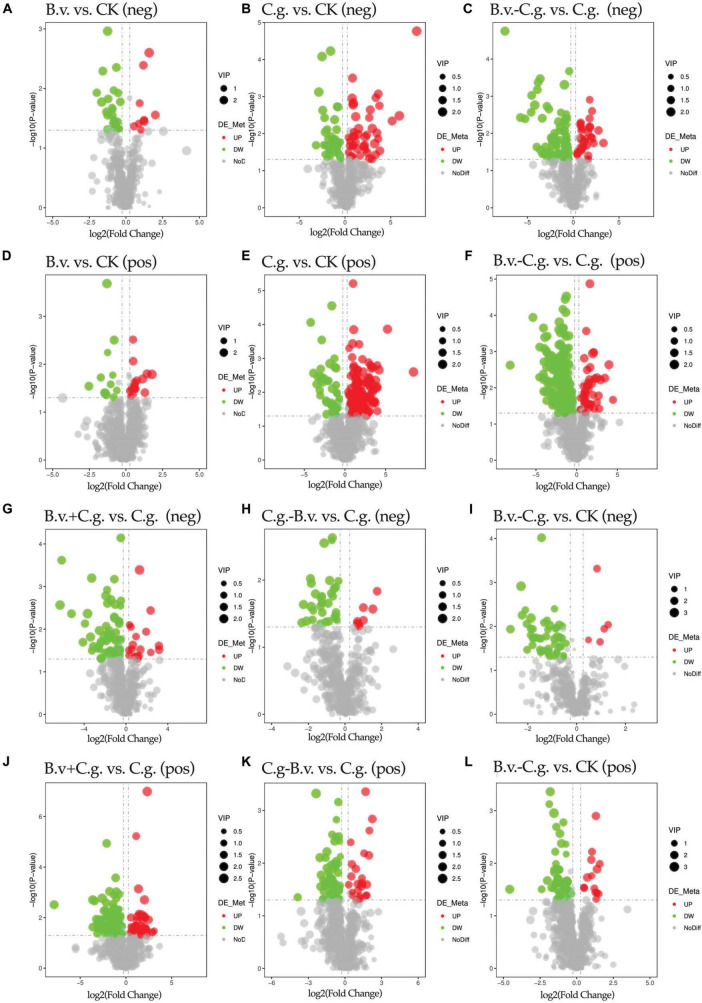
Volcano plot of differential abundance metabolites. Group B.v.: solely inoculated with *B. velezensis*; Group CK: blank control; Group C.g.: solely inoculated with *C. gloeosporioides*; Group B.v.–C.g.: *B. velezensis* first, then *C. gloeosporioides*; Group B.v.+C.g.: simultaneous inoculation; Group C.g.–B.v.: *C. gloeosporioides* first, then *B. velezensis.* **(A,B,C,G,H,I)** Correspond to the NEG mode; **(D,E,F,J,K,L)** Correspond to the POS mode. The *x*-axis represents the log_2_(Fold Change) of metabolites in different groups, and the *y*-axis represents the significance level [-log_10_(*P*-value)] of the differences. Each point in the volcano plot represents a metabolite, with significantly upregulated metabolites shown in red, significantly downregulated metabolites shown in green, and the size of the dots indicating the VIP (Variable Importance in Projection) value.

Volcano plots visually demonstrate the distribution of differential abundance metabolites. The study analyzed six comparison groups: B.v. vs. CK, C.g. vs. CK, B.v.−C.g. vs. C.g., B.v.+C.g. vs. C.g., C.g.−B.v. vs. C.g., and B.v.−C.g. vs. CK (Figure 4). In positive and negative modes, the comparison groups with the highest total numbers of differential abundance metabolites were B.v.−C.g. vs. C.g. (244 and 112), C.g. vs. CK (160 and 84), and E. vs. C (132 and 86), respectively. The groups with the lowest totals were B.v. vs. CK (27 and 32), C.g.−B.v. vs. C.g. (83 and 40), and B.v.−C.g. vs. CK (72 and 55). These results indicate that the B.v. vs. CK group had the fewest differential abundance metabolites in both modes, suggesting a minimal impact of *B. velezensis* application alone on walnut plant metabolism. Furthermore, the B.v.−C.g. vs. C.g. group showed significantly more differential abundance metabolites than C.g. vs. CK, indicating the highest change in metabolite abundance. This suggests that *C. gloeosporioides* infection caused significant metabolic changes in walnut plants, and *B. velezensis* application further enhanced this effect, leading to an increase in differential abundance metabolites. This could potentially aid the host walnut in resisting *C. gloeosporioides* invasion.

**FIGURE 5:**
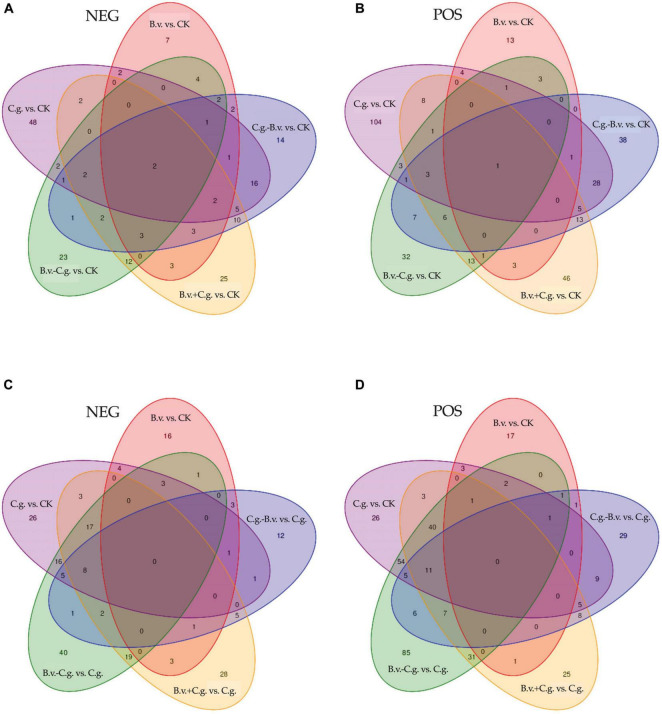
The difference between different treatment groups and control group CK, different treatment groups and treatment group C.g. Venn diagram. **(A,B)** is the difference Venn diagram between different treatment groups and blank control group CK. **(C,D)** is the difference Venn diagram between different treatment groups and treatment group C.g.

Figures 5A, B showed the differential abundance of metabolites between various treatment groups and the control group CK. In the NEG test results, the differential abundance metabolites for B.v. vs. CK, C.g. vs. CK, B.v.−C.g. vs. CK, B.v.+C.g. vs. CK, and C.g.−B.v. vs. CK were 32, 84, 55, 71, and 67, respectively. In the POS test results, these numbers were 27, 160, 72, 100, and 103. The number of differential abundance metabolites between groups and common metabolites varied, indicating distinct metabolic changes. Figures 5C, D compared treatment groups to group C.g. In the NEG test results, the differential abundance metabolites for B.v. vs. CK, C.g. vs. CK, B.v.−C.g. vs. C.g., B.v.+C.g. vs. C.g., and C.g.−B.v. vs. C.g. were 32, 84, 112, 86, and 39, respectively. In the POS test results, these numbers were 27, 160, 244, 132, and 83. These results showed that *B. velezensis* significantly impacted plant metabolic profiles, whether applied alone, before, simultaneously, or after *C. gloeosporioides* inoculation. The highest number of differential abundance metabolites was observed in the *C. gloeosporioides*-treated group C.g. compared to the control group CK and other treatment combinations, especially in the POS test results, indicating a substantial metabolic response to *C. gloeosporioides* infection. Simultaneous or subsequent application of *B. velezensis* and *C. gloeosporioides* resulted in significant metabolic changes, suggesting interactions or metabolic adjustments due to combined stress or biocontrol effects.

#### 3.2.4 Analysis of KEGG enrichment results

**FIGURE 6:**
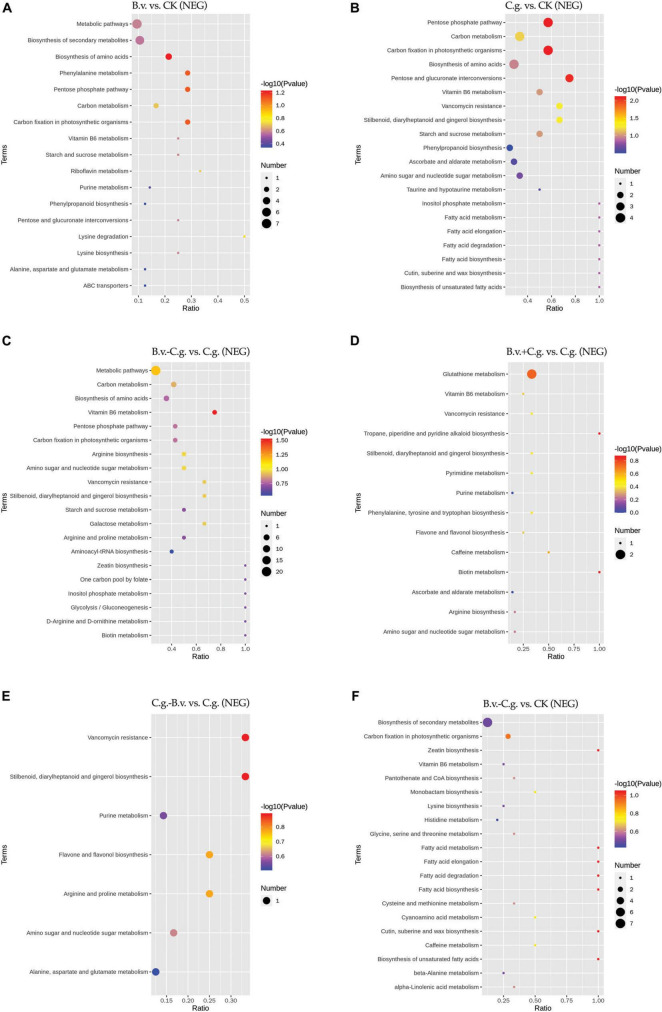
KEGG pathway bubble chart (NEG mode). Group B.v.: solely inoculated with *B. velezensis*; Group CK: blank control; Group C.g.: solely inoculated with *C. gloeosporioides*; Group B.v.–C.g.: *B. velezensis* first, then *C. gloeosporioides*; Group B.v.+C.g.: simultaneous inoculation; Group C.g.–B.v.: *C. gloeosporioides* first, then *B. velezensis*. **(A)**: B.v. vs. CK ; **(B)**: C.g. vs. CK; **(C)**: B.v.-C.g. vs. C.g.; **(D)**: B.v.+C.g. vs. C.g.; **(E)**: C.g.-B.v. vs. C.g.; **(F)**: B.v.-C.g. vs. CK. Each figure presented the top 20 significantly enriched metabolic pathways in the KEGG enrichment analysis. The *x*/*y* axis in the figure represented the number of differential abundance metabolites/total identified metabolites in each pathway, indicating higher enrichment levels with larger values. The color of the points indicated the *P*-value of the hypergeometric test, with smaller values signifying greater reliability and statistical significance. Point size denoted the number of differential abundance metabolites in each pathway, with larger sizes indicating greater differential expression.

**FIGURE 7:**
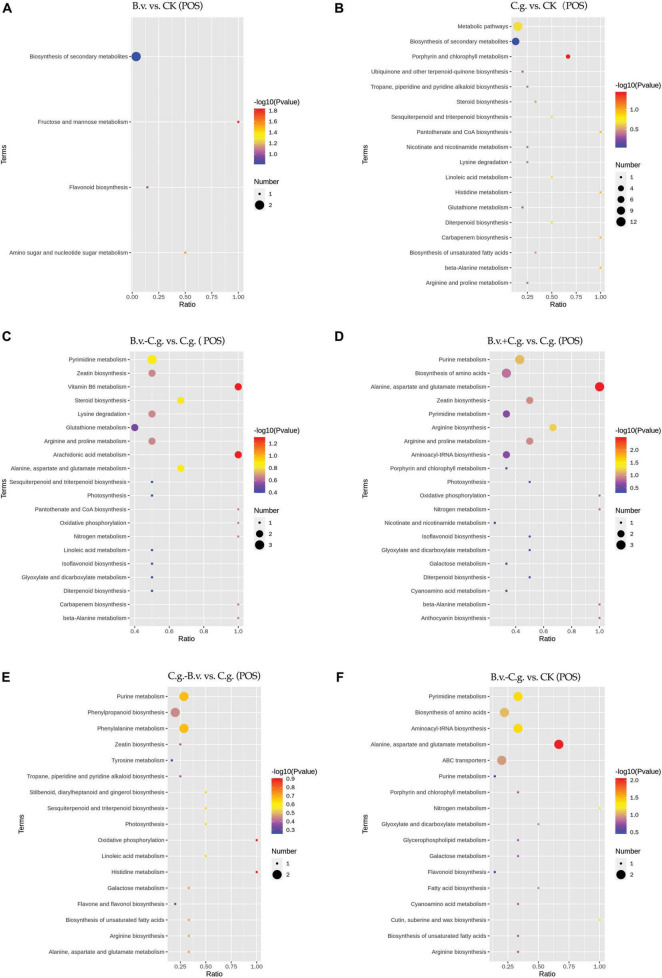
KEGG pathway bubble chart (POS mode). Group B.v.: solely inoculated with *B. velezensis*; Group CK: blank control; Group C.g.: solely inoculated with *C. gloeosporioides*; Group B.v.–C.g.: *B. velezensis* first, then *C. gloeosporioides*; Group B.v.+C.g.: simultaneous inoculation; Group C.g.–B.v.: *C. gloeosporioides* first, then *B. velezensis*. **(A)**: B.v. vs. CK; **(B)**: C.g. vs. CK; **(C)**: B.v.-C.g. vs. C.g.; **(D)**: B.v.+C. g. vs. C.g.; **(E)**: C.g.-B.v. vs. C.g.; **(F)**: B.v.-C.g. vs. CK. Each figure presented the top 20 significantly enriched metabolic pathways in the KEGG enrichment analysis. The *x*/*y* axis in the figure represented the number of differential abundance metabolites/total identified metabolites in each pathway, indicating higher enrichment levels with larger values. The color of the points indicated the *P*-value of the hypergeometric test, with smaller values signifying greater reliability and statistical significance. Point size denoted the number of differential abundance metabolites in each pathway, with larger sizes indicating greater differential expression.

The metabolite enrichment results reveal distinct differences among comparison groups in both negative (Figure 6) and positive (Figure 7) modes. In the B.v. vs. CK group, enriched differential abundance metabolites in negative mode primarily concentrate on metabolic pathways and biosynthesis of secondary metabolites, while in positive mode, the focus remains on the biosynthesis of secondary metabolites. This indicates that biosynthesis of secondary metabolites shows significant variation in both modes within the B.v. vs. CK group, highlighting its crucial regulatory role. In the C.g. vs. CK group, enriched differential abundance metabolites in negative mode span multiple metabolic pathways, including biosynthesis of secondary metabolites, metabolic pathways, the pentose phosphate pathway, photosynthetic carbon fixation, carbon metabolism, and biosynthesis of amino acids. In positive mode, the emphasis is again on metabolic pathways and biosynthesis of secondary metabolites, suggesting that metabolite changes in the C.g. vs. CK group involve various metabolic and biosynthetic pathways. This may imply that walnut plants adjust these pathways in response to stress and environmental changes caused by *C. gloeosporioides* infection. For the B.v.−C.g. vs. C.g. group, differential abundance metabolites enriched in negative mode mainly involve metabolic pathways and biosynthesis of amino acids, while in positive mode, they include arachidonic acid metabolism, vitamin B6 metabolism, steroid biosynthesis, and metabolism of alanine, aspartate, and glutamate among others. This shows that the application of *B. velezensis* induces walnut plants to adjust various metabolic and biosynthetic pathways in response to *C. gloeosporioides* infection. In the B.v.+C.g. vs. C.g. group, glutathione metabolism is predominantly enriched in negative mode, while in positive mode, differential abundance metabolites include metabolism of alanine, aspartate, glutamate, arginine biosynthesis, and purine metabolism. This suggests that glutathione metabolism undergoes significant changes in negative mode, and various metabolic and biosynthetic pathways are affected in positive mode due to *B. velezensis* application, helping to reduce the severity of *C. gloeosporioides* infection. The C.g.−B.v. vs. C.g. group shows fewer enriched differential abundance metabolites in negative mode, but in positive mode, enrichment mainly involves purine metabolism, phenylalanine metabolism, and biosynthesis of phenylpropanoid substances. This indicates a relatively minor difference in this comparison group, yet *B. velezensis* treatment still modulates walnut plant metabolic pathways. In the B.v.−C.g. vs. CK group, differential metabolite enrichment in the negative mode primarily involved the biosynthesis of secondary metabolites and carbon fixation during photosynthesis. This indicated that simultaneous application of *B. velezensis* and *C. gloeosporioides* induced adjustments in walnut leaf secondary metabolism and optimized carbon fixation during photosynthesis, crucial for enhancing plant adaptation to environmental stress and growth efficiency. In the positive mode, enriched metabolites included pathways such as alanine, aspartate, and glutamate metabolism, which likely played a role in regulating processes such as amino acid biosynthesis, pyrimidine metabolism, and aminoacyl-tRNA biosynthesis in walnut leaf tissues. Additionally, pathways like ABC transporters showed enrichment, potentially contributing to enhancing walnut plant resistance against the pathogen *C. gloeosporioides*. Overall, the enrichment results of metabolites in negative and positive modes across different comparison groups demonstrate that walnut plants undergo significant regulatory changes in their metabolic and biosynthetic pathways when infected by *C. gloeosporioides* or treated with *B. velezensis*, enhancing resistance against the pathogen.

### 3.3 Integrative transcriptomic and metabolomic analysis

**FIGURE 8:**
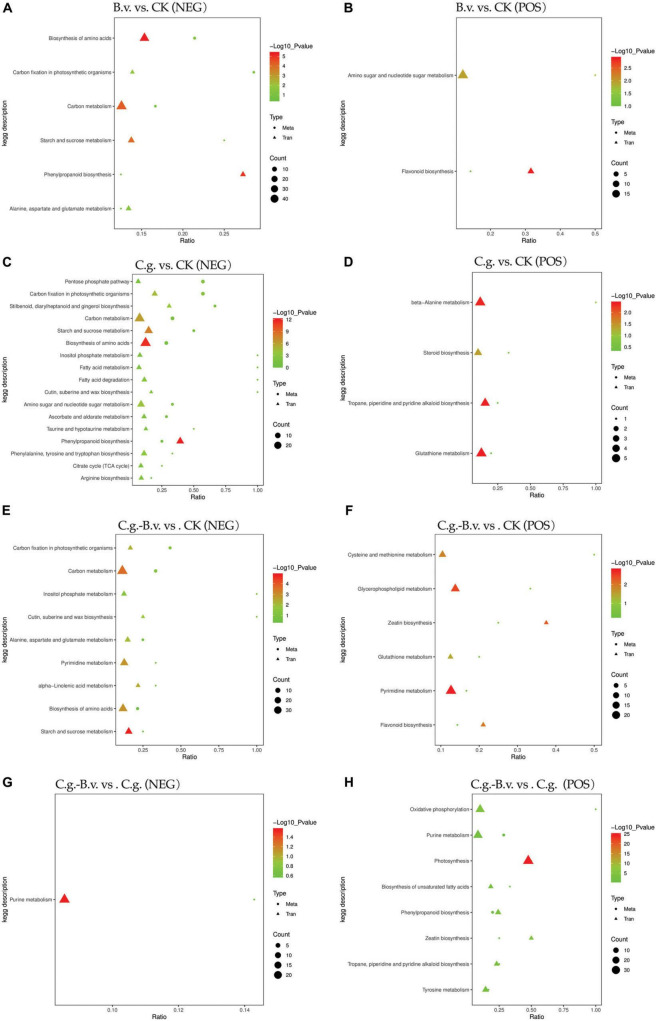
KEGG enrichment bubble plots of differentially expressed genes and differential abundance metabolites. Group B.v.: solely inoculated with *B. velezensis*; Group CK: blank control; Group C.g.: solely inoculated with *C. gloeosporioides*; Group B.v.–C.g.: *B. velezensis* first, then *C. gloeosporioides*; Group B.v.+C.g.: simultaneous inoculation; Group C.g.–B.v.: *C. gloeosporioides* first, then *B. velezensis*. **(A,C,E,G)** represented the neg mode; **(B,D,F,H)** represented the pos mode. The *x*-axis showed the ratio of enriched differential abundance metabolites or differentially expressed genes to annotated metabolites or genes in each pathway (Ratio). The *y*-axis displayed KEGG pathways enriched in both the metabolome and transcriptome. Count indicated the number of enriched metabolites or genes in each pathway. Colors represented *P*-values, with lighter colors indicating smaller, more significant pathway enrichments.

In the four comparison pairs analyzed (Figure 8), the C.g. vs. CK pair showed the highest enrichment of differentially expressed genes and differential abundance metabolites, indicating significant differences between healthy and diseased walnut plants. This suggests that *C. gloeosporioides* infection significantly impacts gene regulation and metabolic pathways in walnut plants, leading to considerable biological differences. Meanwhile, the C.g.−B.v. vs. CK comparison exhibited fewer differentially expressed genes and differential abundance metabolites than the C.g. vs. CK pair, indicating that *B. velezensis*-treated diseased plants have fewer differences compared to healthy plants. This suggests that *B. velezensis* treatment may significantly alter walnut plants, reducing differences between diseased and healthy plants. The B.v. vs. CK pair had the fewest enriched genes and metabolites, suggesting minimal biological differences between *B. velezensis*-treated healthy walnut plants and untreated controls, indicating *B. velezensis* treatment introduces minimal additional variation. In contrast, C.g.−B.v. vs. CK showed more differences in genes and metabolites compared to C.g.−B.v. vs. C.g., reflecting the significant impact of *C. gloeosporioides* infection and *B. velezensis* treatment on walnut plants. The B.v. vs. CK comparison in negative mode showed significant enrichment in amino acid synthesis, carbon fixation in photosynthesis, carbon metabolism, starch and sucrose metabolism, alanine, aspartate, and glutamate metabolism, and phenylpropanoid biosynthesis. In positive mode, significant enrichment was observed in amino sugar and nucleotide sugar metabolism, and flavonoid biosynthesis, indicating changes in key metabolic pathways in *B. velezensis*-treated healthy walnut plants compared to untreated ones. These changes reflect the significant impact of *B. velezensis* treatment on the physiological state and metabolism of walnut plants, affecting growth, photosynthesis, carbon metabolism, amino acid metabolism, and secondary metabolism. In the C.g. vs. CK comparison in negative mode, diseased plants showed significant differences from healthy ones in energy metabolism, photosynthesis, carbon metabolism, starch and sucrose metabolism, and amino acid biosynthesis. In positive mode, differences in beta-alanine metabolism, steroid biosynthesis, alkaloid metabolism, and glutathione metabolism were observed, highlighting the metabolic differences between diseased and healthy walnut plants. After *B. velezensis* treatment in the C.g.−B.v. vs. CK pair, significant enrichment was seen in photosynthesis, carbon metabolism, wax synthesis, inositol phosphate metabolism, amino acid metabolism, purine metabolism, and alpha-linolenic acid metabolism in negative mode. In positive mode, differences in cysteine and methionine metabolism, phospholipid metabolism, zeatin biosynthesis, glutathione metabolism, purine metabolism, and flavonoid biosynthesis were noted, indicating *B. velezensis* treatment’s impact on these pathways. Notably, the C.g. vs. CK and C.g.−B.v. vs. CK pairs shared common enriched pathways in negative mode, such as amino acid biosynthesis, starch and sucrose metabolism, carbon fixation in photosynthesis, and carbon metabolism, and in positive mode, glutathione metabolism was commonly enriched. This suggests common differences in these pathways between diseased and healthy plants and those treated with *B. velezensis*, indicating their importance in disease resistance and post-treatment adjustments. In the C.g.−B.v. vs. C.g. comparison in negative mode, significant enrichment in purine metabolism was observed, highlighting differences in this pathway between *B. velezensis*-treated diseased plants and their diseased counterparts. In positive mode, oxidative phosphorylation, purine metabolism, photosynthesis, unsaturated fatty acid biosynthesis, phenylpropanoid biosynthesis, zeatin biosynthesis, and tyrosine metabolism showed significant differences, indicating potential metabolic adjustments in *B. velezensis*-treated diseased plants compared to diseased ones in these aspects.

Based on the combined analysis results from KEGG, a collection and organization of involved differential abundance metabolites and genes were conducted. Given the extensive number of differential genes involved, only those genes that appeared repeatedly across multiple pathways were selected and displayed, as detailed in Table 1. Among the three comparison pairs analyzed, the C.g. vs. CK pair had the highest number of differential abundance metabolites, indicating the most pronounced differences between the two groups. In the B.v. vs. CK comparison, 8 metabolites were identified with differential abundance alongside 34 key differential genes such as *LOC108985009*, which are implicated in the regulation of these metabolites. The application of *B. velezensis* significantly altered several metabolic pathways, including carbon metabolism, carbon fixation in photosynthesis, and amino acid biosynthesis. Specifically, within the amino sugar and nucleotide sugar metabolism pathway, 18 genes showed differential expression leading to the upregulation of d-mannose 6-phosphate, suggesting an enhancement in cell wall strength and integrity which could improve the plant’s resistance to the anthracnose pathogen, *C. gloeosporioides*. Furthermore, the C.g. vs. CK comparison revealed downregulation of metabolites such as α,α-trehalose, affecting pathways like the pentose phosphate pathway, carbon fixation in photosynthesis, carbon metabolism, and amino acid biosynthesis. This downregulation likely reflects metabolic adjustments wherein walnut plants economize energy and resources to combat pathogenic attacks. Conversely, metabolites such as pipecolic acid were upregulated, signaling an activation of defense mechanisms post-pathogen inoculation that enhance cell wall fortification, synthesize defensive secondary metabolites, and adjust energy metabolism and stress responses. Notably, the increase in levels of palmitic acid and ferulic acid underscores the strengthening of cell wall integrity through improved membrane stability and lignin synthesis, respectively, enhancing resistance to biological invasion. In the C.g.−B.v. vs. C.g. comparison, six differential abundance metabolites showed downregulation while two were upregulated in the C.g.−B.v. group. Downregulated metabolites such as lupinine, linoleic acid, salidroside, isoeugenol, and cyclic AMP, are involved in pathways including tropane, piperidine, and pyridine alkaloid biosynthesis, oxidative phosphorylation, and purine metabolism. This pattern suggests that *B. velezensis* may facilitate physiological optimizations that reduce the consumption of energy and resources, thereby enabling more effective pathogen defense. On the other hand, the upregulation of coumarin and adenosine 5′-diphosphate (ADP) in pathways such as phenylpropanoid biosynthesis and oxidative phosphorylation likely promotes the synthesis of metabolites that enhance disease resistance and photosynthetic efficiency. In particular, coumarin’s involvement in antioxidative processes and ADP’s role in energy transformation and signaling underscore their importance in the plant’s adaptive responses to environmental stress.

**TABLE 1:** Differential metabolite statistics from transcriptomic and metabolomic association analysis.

Comparison group name	Regulation status	Differential abundance metabolites (NEG mode)	Differential abundance metabolites (POS mode)	Differential gene (NEG mode)	Differential gene (POS mode)
B.v. vs. CK	DEM down	D-ribulose-5-phosphate; scopolin; α,α-trehalose; adenylosuccinic acid; D-sedoheptulose 7-phosphate; L-saccharopine	Dihydromyricetin	LOC108985009; LOC108989739; LOC108995063; LOC109007871; LOC108986174; LOC109004608; LOC108982574; LOC108979677; LOC109009364; LOC109002958; LOC108989204; LOC109001012; LOC109006536; LOC108994846; LOC109012505; LOC108979480; LOC109008648	LOC109006259; LOC109014012; LOC109008672; LOC109000742; LOC109012911; LOC109012913; LOC108998095; LOC109006298; LOC109006299; LOC109004165; LOC109004164; LOC109004162; LOC108980812; LOC108999688; LOC108988418; LOC109008231; LOC109010986
	DEM up	/	D-mannose 6-phosphate		
C.g. vs. CK	DEM down	D-erythrose 4-phosphate; D-ribulose-5-phosphate; D-glucose 6-phosphate; α,α-trehalose	/	LOC108985009; LOC109005150; LOC109007785; LOC109010220; LOC109003634; LOC108998278; LOC108996646; LOC108985317; LOC108979963; LOC109004987; LOC108996947; LOC108988942; LOC108999180; LOC108988131; LOC108984074; LOC108981179; LOC108998652; LOC108992456; LOC108980812	LOC108989738; LOC109018267; LOC108987596; LOC108984851; LOC109019460; LOC108988131; LOC108984074; LOC108995469; LOC109009357; LOC108982978; LOC109011808; LOC108981208; LOC108983086; LOC109010098; LOC109002259; LOC109017388; LOC109003474; LOC109002260
	DEM up	D-xylulose 5-phosphate; 5-O-caffeoylquinic acid; Palmitic acid; UDP-N-acetyl-α-D-glucosamine; UDP-N-acetylglucosamine; α-ketoglutaric acid; ferulic acid	Spermine; ergocalciferol; pipecolic acid		
C.g.−B.v. vs. C.g	DEM down	Adenylyl-succinate	Lupinine; linoleic acid; salidroside; isoeugenol; cyclic AMP	LOC108986757; LOC108992092; LOC118347625; LOC108992695; LOC109019734; LOC118347949; LOC109009364; LOC109009682; LOC109002958; LOC108991725; LOC109011733; LOC108989481; LOC109005096	LOC108986757; LOC108992092; LOC118347625; LOC108992695; LOC109019734; LOC118347949; LOC109009364; LOC109009682; LOC109002958; LOC108991725; LOC109011733; LOC108989481; LOC109005096; LOC118348746; LOC109004928; LOC109013250; LOC109018267; LOC108987596; LOC108996646; LOC109012843; LOC109012863; LOC109007996; LOC109007998; LOC108983187
	DEM up	/	Coumarin; adenosine 5′-diphosphate (ADP)		

Group B.v.: solely inoculated with *B. velezensis*; Group CK: blank control; Group C.g.: solely inoculated with *C. gloeosporioides*; Group C.g.−B.v.: *C. gloeosporioides* first, then *B. velezensis*.

## 4 Discussion

*C. gloeosporioides*, a major pathogen in walnut anthracnose, severely impacts the walnut industry and other crops like strawberries and mangoes, underscoring the need for effective management strategies (Embaby et al., 2012; Pérez-Mora et al., 2020; Chen et al., 2021; Fang et al., 2021; Feng et al., 2021; Fan et al., 2023a; Zhang et al., 2023). Traditional chemical controls, while effective, pose environmental and health risks, prompting the exploration of biological alternatives. *Bacillus* species, exhibiting notable antifungal effects against *C. gloeosporioides*, represent viable substitutes to chemical treatments (Han et al., 2015; Wang K. et al., 2021; Papathoti et al., 2022; Vu et al., 2023; Luna-Bulbarela et al., 2024). For example, *B. altitudinis* GS-16 and *B. licheniformis* CTCRI EB12 have shown high efficacy in suppressing *C. gloeosporioides* and protecting crops from anthracnose (Ravindran et al., 2023; Wu Y. et al., 2024). Similarly, *B. velezensis* PW192, isolated from *Lagerstroemia macrocarpa* rhizosphere, displayed significant antifungal activity (Jumpathong et al., 2022). Our study further validates the effectiveness of *B. velezensis* in controlling walnut anthracnose, especially with preemptive applications before infection onset. These insights, supporting previous findings (Wu Y. et al., 2024), highlight *Bacillus* spp. as promising agents for sustainable disease management and agricultural productivity enhancement.

Metabolomic analysis showed that treatment groups B.v., C.g., B.v.−C.g., B.v.+C.g., and C.g.−B.v. had significant changes in metabolites such as flavonoids, terpenoids, and steroids compared to control group CK. Flavonoids are known to enhance plant defense mechanisms. For instance, flavonoids have been linked to increased resistance to stem canker in plants (Li et al., 2021) and enhanced protection against *Penicillium digitatum* in citrus (Luo et al., 2015). Similarly, walnuts increase phenolic and flavonoid contents, which helps strengthen disease resistance by modifying cell wall composition (Martini et al., 2009). The results indicated that, compared to group C.g., groups B.v.−C.g., B.v.+C.g., and C.g.−B.v. showed a positive correlation between the number of differential abundance metabolites and the relative control effect. This suggests that pre-application of *B. velezensis* can induce metabolic changes in walnut plants to a greater extent, thereby reducing the incidence of anthracnose and controlling its development. The comparison between groups B.v.−C.g. vs. C.g. and C.g. vs. CK revealed the highest number of differential abundance metabolites, with group B.v.−C.g. vs. C.g. showing the greatest diversity. These changes encompass basic metabolic pathways and amino acid biosynthesis, extending to arachidonic acid metabolism, vitamin B6 metabolism, steroid biosynthesis, and the diversified metabolism of alanine, asparagine, and glutamate. In plants, amino acids not only serve as building blocks for protein formation but also act as precursors for many plant hormones. Furthermore, certain amino acids, such as proline, enhance the plant’s resistance to stress (Zhao et al., 2020; Gutensohn et al., 2022). Vitamin B6 is closely associated with defense responses. Reduced biosynthesis of Vitamin B6 has been demonstrated to increase plant susceptibility to diseases, as confirmed by Liu et al. (2022).

*Bacillus* species act as elicitors, triggering diverse plant defense mechanisms. These elicitors, recognized by plant cell receptors, activate signal transduction pathways that alter the expression of regulatory factors, leading to enhanced synthesis and accumulation of plant compounds (Zhai et al., 2017; Miladinova-Georgieva et al., 2022). Typically, plants regulate their defense through pathways associated with SA, ET, ABA, and JA. Our analysis identified metabolites associated with the SA, ABA, and JA pathways in all comparison groups, while notably, metabolites related to the ET response were absent. SA, ABA, and jasmonates (JAs), often referred to as stress hormones, regulate plant adaptive responses to unfavorable environmental conditions (Veselova et al., 2022; Wang et al., 2022; Yue et al., 2022; Chen et al., 2023; Pigolev et al., 2023). Specifically, SA can modulate the physiological state of the entire plant-endophyte system, enhancing the biocontrol potential of endophytic strains. This is primarily demonstrated by stimulating the motility of endophytes, regulating plant defense responses, and facilitating easier colonization of endophytes within the plants (Pigolev et al., 2023). In our study, metabolites indicative of SA responses, such as 12-oxo-phytodienoic acid, JA, and isoleucine, were detected across all comparison groups. Except for the B.v.−C.g. vs. C.g. comparison group, where methyl jasmonate was not detected, its presence in other groups indicates methyl jasmonate, the volatile ester of JA, acts as a primary signaling molecule in both abiotic and biotic stress responses. It facilitates intra- and inter-plant communication, regulating defense responses, especially the antioxidant system (Wang Y. et al., 2021). Additionally, the presence of ABA differential abundance metabolites in each comparison group signifies that plants initiated the ABA metabolic pathway for defensive responses. Our study corroborates that *B. velezensis* induces plant defense mechanisms by activating the JA, SA, and ABA metabolic pathways, in line with extensive research findings. For instance, treatment of rice roots with *B. velezensis* YC7010 initiated systemic resistance dependent on JA and SA signaling pathways (Harun-Or-Rashid et al., 2018). Yet, additional studies demonstrate that *Bacillus* spp. can also engage the ET pathway in various plants. For example, *B. cereus* inoculation in tobacco plants led to the observation of numerous differentially expressed genes related to JA, SA, and ET, alongside alterations in ABA signaling by Li et al. (2020). Furthermore, *B. subtilis* 26D and *B. velezensis* HN-Q-8 have been shown to bolster systemic resistance against *Rhopalosiphum padi L*. and *Alternaria solani*, respectively, by activating SA, ABA, and ET signaling pathways, thus enhancing plant defense and growth (Bai et al., 2023; Rumyantsev et al., 2023). These insights suggest that the elicitor role of *Bacillus* is linked to the activation of specific plant defense pathways (Veselova et al., 2022).

Integrating transcriptomic and metabolomic analyses offers a comprehensive method for exploring the molecular mechanisms of plant defense against pathogens (Wu H. et al., 2024). Further integrative transcriptomic and metabolomic analysis in the B.v. vs. CK and C.g. vs. CK comparisons revealed upregulation of key metabolites linked to plant cell wall structure, such as d-mannose 6-phosphate, palmitic acid, and ferulic acid. The plant cell wall serves as the primary barrier against pathogenic invasion, playing a crucial role in sensing and responding to external stresses, thereby stimulating defense mechanisms (Cosgrove, 2005). Palmitic acid, vital for maintaining cell membrane integrity, also enhances the cell wall’s dynamic response to environmental challenges (Houston et al., 2016). Ferulic acid is crucial for lignin synthesis, which bolsters the cell wall’s resistance to pathogens; disruptions in lignin biosynthesis can significantly affect plant defenses (Ma et al., 2017; Ma, 2024). The investigation demonstrated that walnut plants in Group B.v., solely treated with *B. velezensis*, and Group C.g., exclusively exposed to *C. gloeosporioides*, manifested augmented resistance in their cell walls against *C. gloeosporioides*. This enhancement in resistance, linked to ISR, is corroborated by our findings, aligning with pivotal research by Zhukov (2015) and Yadav and Chattopadhyay (2023). Additionally, the C.g. vs. CK comparison revealed an upregulation of antimicrobial metabolites such as demethoxycurcumin and pipecolic acid. Demethoxycurcumin, a curcuminoid, has demonstrated significant antifungal properties (Akter et al., 2018). Pipecolic acid, a lysine catabolite, plays a pivotal role in enhancing plant resistance by regulating systemic acquired resistance and local defense mechanisms, influencing the biosynthesis and signaling pathways of salicylic acid and camalexin (Navarova et al., 2012; Wang P. et al., 2021). Furthermore, the C.g.−B.v. vs. C.g. comparison noted an upregulation in coumarins, contributing to increased disease resistance (Perkowska et al., 2021). These compounds, critical in plant defense, are benzopyrone derivatives synthesized from benzene and pyrone rings, exhibiting strong antifungal activity against pathogens such as *Aspergillus flavus* and *Fusarium graminearum* (Sarkanj et al., 2013; Lončar et al., 2023). This synthesis aids in defending plants against various pathogens (Goy et al., 1993; Gnonlonfin et al., 2012; Sun et al., 2014).

## 5 Conclusion

Walnut trees face a significant threat from anthracnose caused by *C. gloeosporioides*. However, *B. velezensis* treatment shows promise in inhibiting fungal growth. Metabolomic analysis revealed changes in the metabolic profile of walnut plants, while transcriptomic analysis determined the key metabolic pathways involved in walnut’s disease resistance mechanism. Multiple genes regulated by *B. velezensis* contribute to the production of defensive metabolites, enhancing walnut plant resistance to *C. gloeosporioides* infection. Combined transcriptomic and metabolomic analysis indicates that *B. velezensis* treatment enhances the host plant’s stress response and metabolic adjustments, thereby increasing resistance.

## Data Availability

The datasets presented in this study can be found in online repositories. The names of the repository/repositories and accession number(s) can be found in this article/Supplementary material.

## Footnotes

1
https://www.mzcloud.org/

2
https://www.genome.jp/kegg/pathway.html

3
https://hmdb.ca/metabolites

4
http://www.lipidmaps.org/

## References

[B1] Akter J. Amzad Hossain M. Sano A. Takara K. (2018). Antifungal activity of various species and strains of turmeric (*Curcuma* spp.) against *Fusarium solani* sensu lato. *Pharm. Chem. J.* 52 320–325. 10.1007/s11094-018-1815-4

[B2] Ali S. A. M. Sayyed R. Z. Mir M. I. Khan M. Y. Hameeda B. Alkhanani M. F. (2022). Induction of systemic resistance in maize and antibiofilm activity of surfactin from *Bacillus velezensis* MS20. *Front. Microbiol.* 13:879739. 10.3389/fmicb.2022.879739 35615505 PMC9126211

[B3] Armenova N. Petrova P. Gerginova M. Krumova E. Kaynarov D. Velkova L. (2024). *Bacillus velezensis* R22 inhibits the growth of multiple fungal phytopathogens by producing surfactin and four fengycin homologues. *Biotechnol. Biotechnol. EQ* 38:2313072. 10.1080/13102818.2024.2313072

[B4] Bai X. Li Q. Zhang D. Zhao Y. Zhao D. Pan Y. (2023). *Bacillus velezensis* strain HN-Q-8 induced resistance to *Alternaria solani* and stimulated growth of potato plant. *Biology* 12:856. 10.3390/biology12060856 37372140 PMC10295523

[B5] Balderas-Ruíz K. A. Gómez-Guerrero C. I. Trujillo-Roldán M. A. Valdez-Cruz N. A. Aranda-Ocampo S. Juárez A. M. (2021). *Bacillus velezensis* 83 increases productivity and quality of tomato (*Solanum lycopersicum* L.): Pre and postharvest assessment. *Curr. Res. Microb. Sci.* 2:100076. 10.1016/j.crmicr.2021.100076 34841365 PMC8610353

[B6] Beneduzi A. Ambrosini A. Passaglia L. M. P. (2012). Plant growth-promoting rhizobacteria (PGPR): Their potential as antagonists and biocontrol agents. *Genet. Mol. Biol.* 35 1044–1051. 10.1590/s1415-47572012000600020 23411488 PMC3571425

[B7] Chen L. Xie Y. L. Wu X. H. Yang X. Wang T. Peng W. X. (2023). Physiological response of Avena sativa to low-temperature stress is promoted by *Bacillus amyloliquefaciens* GL18 and its functional genes. *Russ. J. Plant Physiol.* 69 1–12. 10.1134/s1021443722601586

[B8] Chen X. H. Koumoutsi A. Scholz R. Schneider K. Vater J. Süssmuth R. (2009). Genome analysis of *Bacillus amyloliquefaciens* FZB42 reveals its potential for biocontrol of plant pathogens. *J. Biotechnol.* 140 27–37. 10.1016/j.jbiotec.2008.10.011 19041913

[B9] Chen X. Wang M. Fu M. Wang G. Xiang K. Liu Q. (2021). Metabolic analysis of phenolic compounds associated with walnut anthracnose. *Sci. Silvae Sin.* 57 71–80. 10.11707/j.1001-7488.20211007

[B10] Chomczynski P. Sacchi N. (1987). Single-step method of RNA isolation by acid guanidinium thiocyanate-phenol-chloroform extraction. *Anal. Biochem.* 162 156–159. 10.1006/abio.1987.9999 2440339

[B11] Chouhan R. Ahmed S. Gandhi S. G. (2023). Over-expression of PR proteins with chitinase activity in transgenic plants for alleviation of fungal pathogenesis. *J. Plant Pathol.* 105 69–81. 10.1007/s42161-022-01226-8

[B12] Cosgrove D. J. (2005). Growth of the plant cell wall. *Nat. Rev. Mol. Cell Biol.* 6 850–861. 10.1038/nrm1746 16261190

[B13] da Silva Junior A. L. Borges ÁV. da Silva H. A. O. Leite I. C. H. L. Alves K. S. de Medeiros L. S. (2023). Lipopeptide-enriched extracts of *Bacillus velezensis* B157 for controlling tomato early blight. *Crop Prot.* 172:106317. 10.1016/j.cropro.2023.106317

[B14] Do T. Q. Nguyen T. T. Dinh V. M. (2023). Application of endophytic bacterium *Bacillus velezensis* BTR11 to control bacterial leaf blight disease and promote rice growth. *Egypt. J. Biol. Pest Control* 33 1–10. 10.1186/s41938-023-00740-w

[B15] Doornbos R. F. Geraats B. P. J. Kuramae E. E. Van Loon L. C. Bakker P. A. H. M. (2011). Effects of jasmonic acid, ethylene, and salicylic acid signaling on the rhizosphere bacterial community of *Arabidopsis thaliana*. *Mol. Plant Microbe Interact.* 24 395–407. 10.1094/mpmi-05-10-0115 21171889

[B16] Embaby E. M. Ragab M. E. Dougdoug K. A. Ahmed R. Zveibil A. Maymon M. (2012). Identification of Colletotrichum acutatum and *C. gloeosporioides* on strawberry in Egypt. *Acta Hortic.* 926:657. 10.17660/ActaHortic.2012.926.95

[B17] Fan Y. Guo F. Wu R. Chen Z. Li Z. (2023a). First report of *Colletotrichum gloeosporioides* causing anthracnose on grapevine (*Vitis vinifera*) in Shaanxi province, China. *Plant Dis.* 105:1193. 10.1094/pdis-10-22-2385-pdn 36691266

[B18] Fan Y. He X. Dai J. Yang N. Jiang Q. Xu Z. (2023b). Induced resistance mechanism of *Bacillus velezensis* S3-1 against pepper wilt. *Curr. Microbiol.* 80:367. 10.1007/s00284-023-03470-2 37819393

[B19] Fang H. C. Liu X. Dong Y. Feng S. Zhou R. Wang C. (2021). Transcriptome and proteome analysis of walnut (*Juglans regia* L.) fruit in response to infection by *Colletotrichum gloeosporioides*. *BMC Plant Biol.* 21:249. 10.1186/s12870-021-03042-1 34059002 PMC8166054

[B20] Feng S. Fang H. Liu X. Dong Y. Wang Q. Yang K. Q. (2021). Genome-wide identification and characterization of long non-coding RNAs conferring resistance to *Colletotrichum gloeosporioides* in walnut (*Juglans regia*). *BMC Genom.* 22:15. 10.1186/s12864-020-07310-6 33407106 PMC7789297

[B21] Ferrusquía-Jiménez N. I. González-Arias B. Rosales A. Esquivel K. Escamilla-Silva E. M. Ortega-Torres A. E. (2022). Elicitation of *Bacillus cereus* -Amazcala (B.c -A) with SiO2 nanoparticles improves its role as a plant growth-promoting bacteria (PGPB) in chili pepper plants. *Plants* 11:3445. 10.3390/plants11243445 36559556 PMC9781252

[B22] Fessia A. Ponzio R. Arcibia L. Barros G. Nesci A. (2024). Effects of different light wavelengths on *Bacillus subtilis* and *Bacillus velezensis*, two biocontrol agents isolated from the maize phyllosphere. *Arch. Microbiol.* 206:104. 10.1007/s00203-024-03836-5 38363376

[B23] Fu H. Marian M. Enomoto T. Hieno A. Ina H. Suga H. (2020). Biocontrol of tomato bacterial wilt by foliar spray application of a novel strain of endophytic *Bacillus* sp. *Microb. Environ.* 35 1–11. 10.1264/jsme2.ME20078 33012743 PMC7734409

[B24] Gnonlonfin G. J. B. Sanni A. Brimer L. (2012). Review scopoletin – a coumarin phytoalexin with me-dicinal properties. *Crit. Rev. Plant Sci.* 31 47–56. 10.1080/07352689.2011.616039

[B25] Goy P. A. Signer H. Reist R. Aichholz R. Blum W. Schmidt E. (1993). Accumulation of scopoletin is associated with the high disease resistance of the hybrid *Nicotiana glutinosa* x *Nicotiana* deb-neyi. *Planta* 191:200. 10.1007/bf00199750

[B26] Guo Q. Li Y. Lou Y. Shi M. Jiang Y. Zhou J. (2019). *Bacillus amyloliquefaciens* Ba13 induces plant systemic resistance and improves rhizosphere microecology against tomato yellow leaf curl virus disease. *Appl. Soil Ecol.* 137 154–166. 10.1016/j.apsoil.2019.01.015

[B27] Gutensohn M. Hartzell E. Dudareva N. (2022). Another level of complex-ity: The role of metabolic channeling and metabolons in plant terpenoid metabolism. *Front. Plant Sci.* 13:954083. 10.3389/fpls.2022.954083 36035727 PMC9399743

[B28] Han J. H. Shim H. Shin J. H. Kim K. S. (2015). Antagonistic activities of *Bacillus* spp. strains isolated from tidal flat sediment towards anthracnose pathogens *Colletotrichum acutatum* and *C. gloeosporioides* in South Korea. *Plant Pathol. J.* 31 165–175. 10.5423/ppj.Oa.03.2015.0036 26060435 PMC4453997

[B29] Harun-Or-Rashid M. Kim H. J. Yeom S. I. Yu H. A. Manir M. M. Moon S. S. (2018). *Bacillus velezensis* YC7010 enhances plant defenses against brown planthopper through transcriptomic and metabolic changes in rice. *Front. Plant Sci.* 9:1904. 10.3389/fpls.2018.01904 30622550 PMC6308211

[B30] He D. C. He M. H. Amalin D. M. Liu W. Alvindia D. G. Zhan J. (2021). Biological control of plant diseases: An evolutionary and eco-economic consideration. *Pathogens* 10:1311. 10.3390/pathogens10101311 34684260 PMC8541133

[B31] Hossain M. M. Sultana F. Hyakumachi M. (2017). Role of ethylene signalling in growth and systemic resistance induction by the plant growth-promoting fungus *Penicillium viridicatum* in *Arabidopsis*. *J. Phyto Pathol.* 165 432–441. 10.1111/jph.12577

[B32] Houston K. Tucker M. R. Chowdhury J. Shirley N. Little A. (2016). The plant cell wall: A complex and dynamic structure as revealed by the responses of genes under stress conditions. *Front. Plant Sci.* 7:984. 10.3389/fpls.2016.00984 27559336 PMC4978735

[B33] Ji Z. L. Peng S. Zhu W. Dong J. P. Zhu F. (2020). Induced resistance in nectarine fruit by *Bacillus licheniformis* W10 for the control of brown rot caused by *Monilinia fructicola*. *Food Microbiol.* 92:103558. 10.1016/j.fm.2020.103558 32950152

[B34] Jiao R. Munir S. He P. Yang H. Wu Y. Wang J. (2020). Biocontrol potential of the endophytic *Bacillus amyloliquefaciens* YN201732 against tobacco powdery mildew and its growth promotion. *Biol. Control* 143:104160. 10.1016/j.biocontrol.2019.104160

[B35] Jumpathong W. Intra B. Euanorasetr J. Wanapaisan P. (2022). Biosurfactant-producing *Bacillus velezensis* PW192 as an anti-fungal biocontrol agent against *Colletotrichum gloeosporioides* and *Colletotrichum musae*. *Microorganisms* 10:1017. 10.3390/microorganisms10051017 35630461 PMC9146131

[B36] Li P. Ruan Z. Fei Z. Yan J. Tang G. (2021). Integrated transcriptome and metabolome analysis revealed that flavonoid biosynthesis may dominate the resistance of *Zanthoxylum bungeanum* against stem canker. *J. Agric. Food Chem.* 69 6360–6378. 10.1021/acs.jafc.1c00357 34043342

[B37] Li Y. Zhao M. Chen W. Du H. Xie X. Wang D. (2020). Comparative transcriptomic analysis reveals that multiple hormone signal transduction and carbohydrate metabolic pathways are affected by Bacillus cereus in *Nicotiana tabacum*. *Genomics* 112 4254–4267. 10.1016/j.ygeno.2020.07.022 32679071

[B38] Liu H. Lu C. Li Y. Wu T. Zhang B. Liu B. (2022). The bacterial effector AvrRxo1 inhibits vitamin B6 biosynthesis to promote infection in rice. *Plant Commun.* 3:100324. 10.1016/j.xplc.2022.100324 35576156 PMC9251433

[B39] Lončar M. Gašo-Sokač D. Molnar M. (2023). Coumarin derivatives as antifungal agents-A review. *Czech J. Food Sci.* 41 79–91. 10.17221/178/2021-cjfs

[B40] Luna-Bulbarela A. Romero-Gutiérrez M. T. Tinoco-Valencia R. Ortiz E. Martínez-Romero M. E. Galindo E. (2024). Response of *Bacillus velezensis* 83 to interaction with *Colletotrichum gloeosporioides* resembles a Greek phalanx-style formation: A stress resistant phenotype with antibiosis capacity. *Microbiol. Res.* 280:127592. 10.1016/j.micres.2023.127592 38199003

[B41] Luo T. Xu K. Y. Luo Y. Chen J. J. Sheng L. Wang J. Q. (2015). Distinct carotenoid and flavonoid accumulation in a spontaneous mutant of ponkan (citrus reticulata blanco) results in yellowish fruit and enhanced postharvest resistance. *J. Agric. Food Chem.* 63 8601–8614. 10.1021/acs.jafc.5b02807 26329679

[B42] Ma Q. H. (2024). Lignin biosynthesis and its diversified roles in disease resistance. *Genes (Basel)* 15:295. 10.3390/genes15030295 38540353 PMC10969841

[B43] Ma Q. H. Zhu H. H. Qiao M. Y. (2017). Contribution of both lignin content and sinapyl monomer to disease resistance in tobacco. *Plant Pathol.* 67 642–650. 10.1111/ppa.12767

[B44] Martini S. D’Addario C. Colacevich A. Focardi S. Borghini F. Santucci A. (2009). Antimicrobial activity against *Helicobacter pylori* strains and antioxidant properties of blackberry leaves (*Rubus ulmifolius*) and isolated compounds. *Int. J. Antimicrob. Agents* 34 50–59. 10.1016/j.ijantimicag.2009.01.010 19386474

[B45] Miladinova-Georgieva K. Geneva M. Stancheva I. Petrova M. Sichanova M. Kirova E. (2022). Effects of different elicitors on micropropagation, biomass and secondary metabolite production of stevia rebaudiana bertoni-a review. *Plants* 12:153. 10.3390/plants12010153 36616282 PMC9824860

[B46] Navarova H. Bernsdorff F. Doring A. C. (2012). Pipecolic acid, an endogenous mediator of defense amplification and priming, is a critical regulator of inducible plant immunity. *Plant Cell* 24 5123–5141. 10.1105/tpc.112.103564 23221596 PMC3556979

[B47] Nie P. P. Li X. Wang S. N. Guo J. H. Zhao H. W. Niu D. D. (2017). Induced systemic resistance against *Botrytis cinerea* by *Bacillus cereus* AR156 through a JA/ET- and NPR1-dependent signaling pathway and activates PAMP-triggered immunity in Arabidopsis. *Front. Plant Sci.* 8:238. 10.3389/fpls.2017.00238 28293243 PMC5329000

[B48] Papathoti N. K. Mendam K. Kanduri B. H. S. Thepbandit W. Sangpueak R. Saengchan C. (2022). Investigation of bioactive com-pounds from *Bacillus* sp. against protein homologs CDC42 of *Colletotrichum gloeosporioides* causing anthrac-nose disease in cassava by using molecular docking and dynamics studies. *Front. Mol. Biosci.* 9:1010603. 10.3389/fmolb.2022.1010603 36213126 PMC9537347

[B49] Pérez-Mora J. L. Mora-Romero G. A. Beltrán-Peña H. García-León E. Lima N. B. Camacho-Tapia M. (2020). First report of *Colletotrichum siamense* and *C. gloeosporioides* causing anthracnose of *Citrus* spp. in Mexico. *Plant Dis.* 105:496. 10.1094/pdis-08-20-1743-pdn 32910725

[B50] Perkowska I. Potrykus M. Siwinska J. Siudem D. Lojkowska E. Ihnatowicz A. (2021). Interplay between coumarin accumulation, iron deficiency and plant resistance to *Dickeya* spp. *Int. J. Mol. Sci.* 22:6449. 10.3390/ijms22126449 34208600 PMC8235353

[B51] Pieterse C. M. van Wees S. C. van Pelt J. A. Knoester M. Laan R. Gerrits H. (1998). A novel signaling pathway controlling induced systemic resistance in *Arabidopsis*. *Plant Cell* 10 1571–1580. 10.1105/tpc.10.9.1571 9724702 PMC144073

[B52] Pieterse C. Zamioudis C. Berendsen R. L. Weller D. M. Van Wees S. C. M. Bakker P. A. H. M. (2014). Induced systemic resistance by beneficial microbes. *Annu. Rev. Phytopathol.* 52 347–375. 10.1146/annurev-phyto-082712-102340 24906124

[B53] Pigolev A. V. Degtyaryov E. A. Miroshnichenko D. N. Savchenko T. V. (2023). Prospects for the application of jasmonates, salicylates, and abscisic acid in agriculture to increase plant stress resistance (review) Sel’sk. *Biologiya* 58 3–22. 10.15389/agrobiology.2023.1.3rus

[B54] Rahman V. Meena K. R. Al-Ani L. K. T. Singh A. Kumar A. (2023). *Bacillus velezensis* strain improvement to control *Helminthosporium maydis* causing southern corn leaf blight disease in maize. *Eur. J. Plant Pathol.* 167 301–313. 10.1007/s10658-023-02708-w

[B55] Rania A. B. A. Catalina S. Constantine G. Ahlem N. Hayfa J. K. Papadopoulou K. K. (2017). Involvement of lipopeptide antibiotics and chitinase genes and induction of host defense in suppression of *Fusarium* wilt by endophytic *Bacillus* spp. in tomato. *Crop Prot.* 99 45–58. 10.1016/j.cropro.2017.05.008

[B56] Rao G. Sui J. Zhang J. (2016). Metabolomics reveals significant variations in metabolites and correlations regarding the maturation of walnuts (*Juglans regia* L.). *Biol. Open* 5 829–836. 10.1242/bio.017863 27215321 PMC4920193

[B57] Ravindran A. P. Lajapathy J. M. Lalithakumari S. G. Mohan A. K. Cyriac T. Usha S. S. (2023). Efficacy of Bacillus licheniformis: A biocontrol agent against *Colletotrichum gloeosporioides* Penz. (Penz. & Sacc.) causing anthracnose in greater yam (*Dioscorea alata* L.). *Egypt J. Biol. Pest Control* 33 1–12. 10.1186/s41938-023-00755-3

[B58] Romera F. J. García M. J. Lucena C. Martínez-Medina A. Aparicio M. A. Ramos J. (2019). Induced systemic resistance (ISR) and fe deficiency responses in dicot plants. *Front. Plant Sci.* 10:287. 10.3389/fpls.2019.00287 30915094 PMC6421314

[B59] Rumyantsev S. D. Veselova S. V. Burkhanova G. F. Alekseev V. Y. Maksimov I. V. (2023). *Bacillus subtilis* 26D triggers induced systemic resistance against *Rhopalosiphum padi* L. by regulating the expression of genes Ago, Dcl and microRNA in bread spring wheat. *Microorganisms* 11:2983. 10.3390/microorganisms11122983 38138127 PMC10745712

[B60] Russi A. Granada C. E. Schwambach J. (2024). Optimization of *Bacillus velezensis* S26 sporulation for enhanced biocontrol of gray mold and anthracnose in postharvest strawberries. *Postharvest Biol. Technol.* 210:112737. 10.1016/j.postharvbio.2023.112737

[B61] Ryu C. M. Farag M. A. Hu C. H. Reddy M. S. Kloepper J. W. Paré P. W. (2004). Bacterial volatiles induce systemic resistance in *Arabidopsis*. *Plant Physiol.* 134 1017–1026. 10.1104/pp.103.026583 14976231 PMC389924

[B62] Sahebani N. Omranzade F. (2020). Assessment of plant defence induction and biocontrol potential of *Bacillus megaterium* wr101 against *Meloidogyne javanica*. *Nematology* 22 1091–1099. 10.1163/15685411-bja10013

[B63] Samaniego-Gámez B. Y. Garruña R. Tun-Suárez J. M. Moreno-Valenzuela O. A. Reyes-Ramírez A. Valle-Gough R. E. (2021). Healthy photosynthetic mechanism suggests ISR elicited by *Bacillus* spp. in *Capsicum* Chinense plants infected with PepGMV. *Pathogens* 10:455. 10.3390/pathogens10040455 33920312 PMC8069211

[B64] Sarkanj B. Molnar M. Cacic M. Gille L. (2013). 4-Methyl-7-hydroxycoumarin antifungal and antioxidant activity enhancement by substitution with thiosemicarbazide and thiazolidinone moieties. *Food Chem.* 139 488–495. 10.1016/j.foodchem.2013.01.027 23561135

[B65] Saxena A. K. Kumar M. Chakdar H. Anuroopa N. Bagyaraj D. J. (2020). Bacillus species in soil as a natural resource for plant health and nutrition. *J. Appl. Microbiol.* 128 1583–1594. 10.1111/jam.14506 31705597

[B66] Sukkasem P. Kurniawan A. Kao T. C. Chuang H. W. (2018). A multifaceted rhizobacterium *Bacillus licheniformis* functions as a fungal antagonist and a promoter of plant growth and abiotic stress tolerance. *Environ. Exp. Bot.* 155 541–551. 10.1016/j.envexpbot.2018.08.005

[B67] Sun H. Wang L. Zhang B. Ma J. Hettenhausen C. Cao G. (2014). Scopoletin is a phytoalexin against *Alternaria alternata* in wild tobacco dependent on jasmonate signalling. *J. Exp. Bot.* 65 4305–4315. 10.1093/jxb/eru203 24821958 PMC4112635

[B68] Tang Z. Cao X. Zhang H. (2023). Production of iturin A by *Bacillus velezensis* ND and its biological control characteristics. *J. Basic Microbiol.* 63 179–189. 10.1002/jobm.202200473 36515292

[B69] Veselova S. V. Sorokan A. V. Burkhanova G. F. Rumyantsev S. D. Cherepanova E. A. Alekseev V. Y. (2022). By modulating the hormonal balance and ribonuclease activity of tomato plants *Bacillus subtilis* induces defense response against potato virus x and potato virus Y. *Biomolecules* 12:288. 10.3390/biom12020288 35204789 PMC8961569

[B70] Vu T. X. Tran T. B. Tran M. B. Do T. T. K. Do L. M. Dinh M. T. (2023). Efficient control of the fungal pathogens *Colletotrichum gloeosporioides* and *Penicillium digitatum* infecting citrus fruits by native soilborne *Bacillus velezensis* strains. *Heliyon* 9:e13663. 10.1016/j.heliyon.2023.e13663 36852059 PMC9958435

[B71] Wang J. Peng Y. Xie S. Yu X. Bian C. Wu H. (2023). Biocontrol and molecular characterization of *Bacillus velezensis* D against tobacco bacterial wilt. *Phytopathol. Res.* 5 1–14. 10.1186/s42483-023-00204-x

[B72] Wang K. Qin Z. Wu S. Zhao P. Zhen C. Gao H. (2021). Antifungal mechanism of volatile organic compounds produced by *Bacillus subtilis* CF-3 on *Colletotrichum gloeosporioides* assessed using omics technology. *J. Agric. Food Chem.* 69 5267–5278. 10.1021/acs.jafc.1c00640 33899461

[B73] Wang P. Luo Q. Yang W. Ahammed G. J. Ding S. Chen X. (2021). A novel role of pipecolic acid biosynthetic pathway in drought tolerance through the antioxidant system in tomato. *Antioxidants (Basel)* 10:1923. 10.3390/antiox10121923 34943026 PMC8750784

[B74] Wang Y. Mostafa S. Zeng W. Jin B. (2021). Function and mechanism of jasmonic acid in plant responses to abiotic and biotic stresses. *Int. J. Mol. Sci.* 22:8568. 10.3390/ijms22168568 34445272 PMC8395333

[B75] Wang K. Qin Z. Wu S. Zhao P. Zhen C. Gao H. (2022). *Bacillus amyloliquefaciens* GB03 augmented tall fescue growth by regulating phytohormone and nutrient homeostasis under nitrogen deficiency. *Front. Plant Sci.* 13:979883. 10.3389/fpls.2022.979883 36275534 PMC9582836

[B76] Wang L. M. Zhu T. H. (2023). Strong opponent of walnut anthracnose-*Bacillus velezensis* and its transcriptome analysis. *Microorganisms* 11:11081885. 10.3390/microorganisms11081885 37630445 PMC10456653

[B77] Wang Q. Ji Y. Qu Y. Qi Y. Li D. Liu Z. (2020). The response strategies of *Colletotrichum gloeosporioides* s.s. due to the stress caused by biological control agent *Bacillus amyloliquefaciens* deciphered by transcriptome analyses. *Biol. Control* 150:104372. 10.1016/j.biocontrol.2020.104372

[B78] Wang X. Wang R. He S. Weng Y. Lan B. Zhou L. (2024). Biocontrol potential of *Bacillus velezensis* wr8 secondary metabolites against *Penicillium* sp. *Gene* 892:147872. 10.1016/j.gene.2023.147872 37802404

[B79] Want E. J. Masson P. Michopoulos F. Wilson I. D. Theodoridis G. Plumb R. S. (2013). Global metabolic profiling of animal and human tissues via UPLC-MS. *Nat. Protoc.* 8 17–32. 10.1038/nprot.2012.135 23222455

[B80] Wei J. Zhao J. Suo M. Wu H. Zhao M. Yang H. (2023). Biocontrol mechanisms of *Bacillus velezensis* against *Fusarium oxysporum* from Panax ginseng. *Biol. Control* 182:105222. 10.1016/j.biocontrol.2023.105222

[B81] Wen B. Mei Z. L. Zeng C. W. Liu S. Q. (2017). metaX: A flexible and comprehensive software for processing metabolomics data. *BMC Bioinform.* 18:1–14. 10.1186/s12859-017-1579-y 28327092 PMC5361702

[B82] Wu G. W. Liu Y. P. Xu Y. Zhang G. S. Shen Q. R. Zhang R. F. (2018). Exploring elicitors of the beneficial rhizobacterium *Bacillus amyloliquefaciens* SQR9 to induce plant systemic resistance and their interactions with plant signaling pathways. *MPMI* 31 560–567. 10.1094/mpmi-11-17-0273-r 29309236

[B83] Wu H. Sun Y. Ma L. Cheng S. Lv D. Hao J. (2024). Microbial exopolysaccharide EPS66A inducing walnut (*Juglans regia*) resistance to bacterial blight. *Food Chem.* 435:137551. 10.1016/j.foodchem.2023.137551 37801767

[B84] Wu Y. Tan Y. Peng Q. Xiao Y. Xie J. Li Z. (2024). Biocontrol potential of endophytic bacterium *Bacillus altitudinis* GS-16 against tea anthracnose caused by *Colletotrichum gloeosporioides*. *PeerJ.* 12:e16761. 10.7717/peerj.16761 38223761 PMC10785793

[B85] Yadav S. Chattopadhyay D. (2023). Lignin: The building block of defense responses to stress in plants. *J. Plant Growth Regul.* 42 6652–6666. 10.1007/s00344-023-10926-z

[B86] Yue L. Uwaremwe C. Tian Y. Liu Y. Zhao X. Zhou Q. (2022). *Bacillus amyloliquefaciens* rescues glycyrrhizic acid loss under drought stress in glycyrrhiza uralensis by activating the jasmonic acid pathway. *Front. Microbiol.* 12:798525. 10.3389/fmicb.2021.798525 35368293 PMC8966401

[B87] Zhai X. Jia M. Chen L. Zheng C. J. Rahman K. Han T. (2017). The regulatory mechanism of fungal elicitor-induced secondary metabolite biosynthesis in medical plants. *Crit. Rev. Microbiol.* 43 238–261. 10.1080/1040841x.2016.1201041 27936989

[B88] Zhang M. Xiao C. Tan Q. Dong L. Liu X. Pu J. (2023). The involvement of the laccase gene Cglac13 in mycelial growth, germ tube development, and the pathogenicity of *Colletotrichum gloeosporioides* from Mangoes. *J. Fungi* 9:503. 10.3390/jof9050503 37233214 PMC10219046

[B89] Zhang S. Reddy M. S. Kloepper J. W. (2002). Development of assays for assessing induced systemic resistance by plant growth-promoting rhizobacteria against blue mold of tobacco. *Biol. Control* 23 79–86. 10.1006/bcon.2001.0992

[B90] Zhao Y. Cui J. Liu M. Y. Zhao L. (2020). Progress on terpenoids with biological activities produced by plant endophytic fungi in china between 2017 and 2019. *Nat. Prod. Commun.* 15 1–18. 10.1177/1934578x20937204

[B91] Zhou Z. Tang X. Peng L. Ding H. (2023). Complete genome sequence of *Bacillus velezensis* GUAL210, a potential biocontrol agent isolated from pepper rhizosphere. *Plant Dis.* 107 915–918. 10.1094/PDIS-07-22-1585-A 36265149

[B92] Zhukov A. V. (2015). Palmitic acid and its role in the structure and functions of plant cell membranes. *Russ. J. Plant Physiol.* 62 706–713. 10.1134/s1021443715050192

