# Supplementary material for: Combined transcriptomic and metabolomic analysis of the mechanism by which Bacillus velezensis induces resistance to anthracnose in walnut
Source: Front Microbiol. 2024 Oct 9;15:1420922. doi: 10.3389/fmicb.2024.1420922 (PMC11496756; doi:10.3389/fmicb.2024.1420922)
Supplement: Supplementary file 1 [file Data_Sheet_1.docx]

Table S1 Potted plant experimental treatment methods and schedule

| Treatment group  Time | Group A | Group B | Group C | Group D | Group E | Group H |
| --- | --- | --- | --- | --- | --- | --- |
| 1d | BV | SW | SW | BV | SW | SW |
| 2d |  |  |  |  |  |  |
| 3d |  |  |  |  |  |  |
| 4d | BV | SW | SW | BV | SW | SW |
| 5d |  |  |  |  |  |  |
| 6d |  |  |  |  |  |  |
| 7d | BV | SW | GC+ SW | GC+BV | GC+BV | GC+ SW |
| 8d |  |  |  |  |  |  |
| 9d |  |  |  |  |  |  |
| 10d | BV | SW | SW | BV | BV | SW |
| 11d |  |  |  |  |  |  |
| 12d |  |  |  |  |  |  |
| 13d | SW | SW | SW | SW | BV | BV |
| 14d |  |  |  |  |  |  |
| 15d |  |  |  |  |  |  |
| 16d | SW | SW | SW | SW | BV | BV |
| 17d |  |  |  |  |  |  |
| 18d |  |  |  |  |  |  |
| 19d | SW | SW | SW | SW | SW | BV |
| 20d |  |  |  |  |  |  |
| 21d |  |  |  |  |  |  |
| 22d | SW | SW | SW | SW | SW | BV |
| 23d |  |  |  |  |  |  |
| 24d |  |  |  |  |  |  |
| 25d | SW | SW | SW | SW | SW | SW |

Note: BV denotes treatment with *B. velezensis* spore fermentation broth at 100 times the concentration. SW indicates sterile water. GC signifies inoculation with *C. gloeosporioides*. Spraying involves a foliar spray combined with root irrigation method, with each walnut tree receiving 500 mL of fermentation broth. Maintain consistent spray pressure and particle size to ensure even coverage of walnut leaves and stems without dripping. The application method for sterile water is identical to this process.


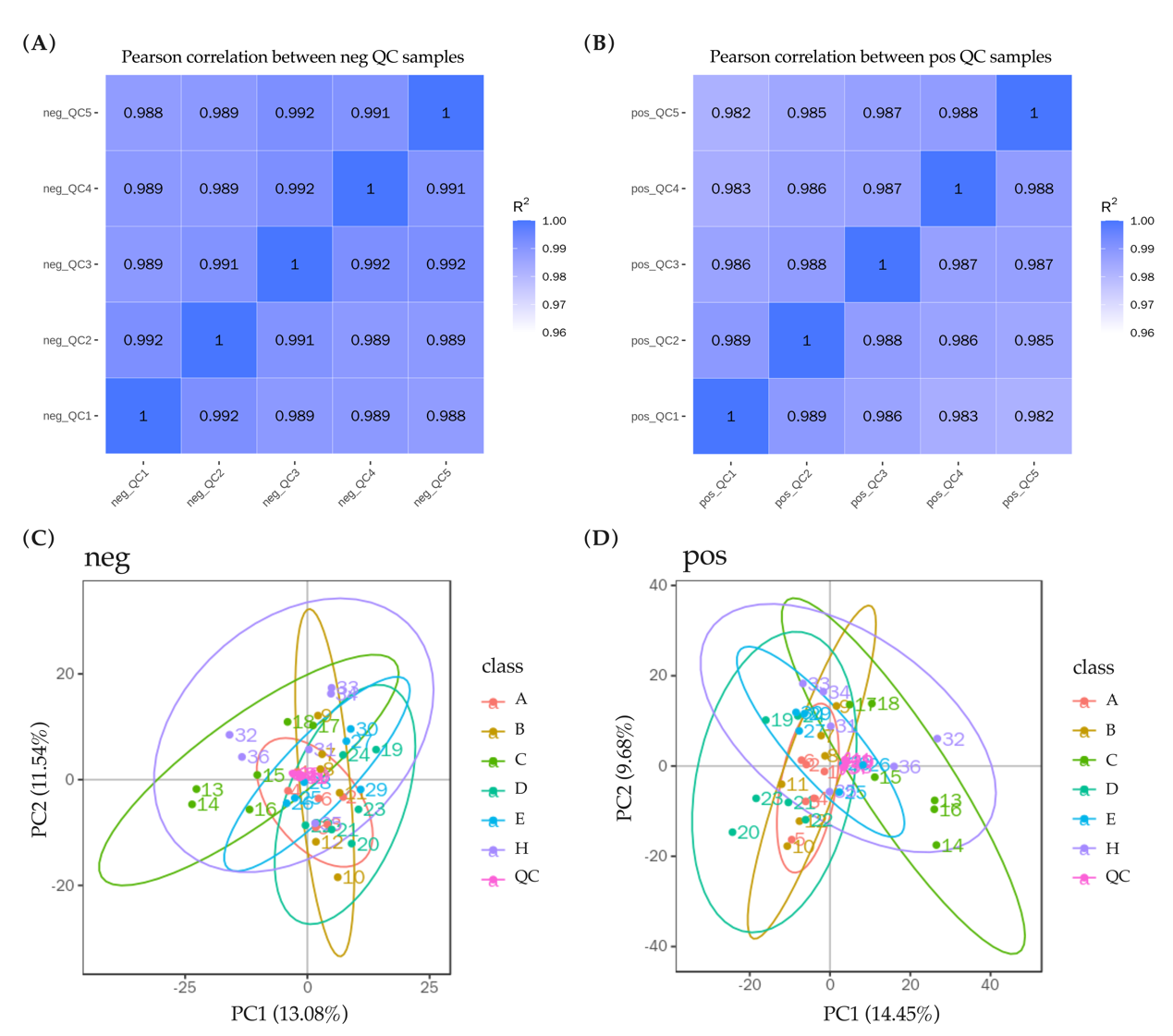
Supplementary Figure S1

**Figure S1.** Data quality control chart. Note: (**A**,**B**): QC sample correlation analysis. (**C,D**): Overall PCA plot of QC and all metabolic samples.
